# Rapid and robust immune response boosting with potent, next-generation adjuvant in viral vector-primed primates

**DOI:** 10.1128/jvi.00389-26

**Published:** 2026-07-27

**Authors:** Hannah A. D. King, Caroline Subra, Emily Tourtellott, Isabella Swafford, Shraddha Basu, Matthew Rossman, Lana Phillips, Nyerhovwo Obarorakpor, Shilei Ding, Maria Grazia Ferrari, Zoltan Beck, Eric Lewitus, Hongjun Bai, Ming Dong, Bonnie M. Slike, Dohoon Kim, Vincent Dussupt, Letzibeth Mendez-Rivera, Nathan Eisel, Lu Zhang, LaTonya D. Williams, Georgia D. Tomaras, Andrés Finzi, Gary R. Matyas, Shelly J. Krebs, Morgane Rolland, Mangala Rao, Dominic Paquin-Proulx, Diane L. Bolton

**Affiliations:** 1 U.S. Military HIV Research Program, Center for Infectious Disease Research, Walter Reed Army Institute of Research 8394, Silver Spring, Maryland, USA; 2 Henry M. Jackson Foundation for the Advancement of Military Medicine 44069, Bethesda, Maryland, USA; 3 Vaccine Research Center, National Institutes of Health 2511, Bethesda, Maryland, USA; 4 Centre de Recherche du CHUM 177460, Montreal, Quebec, Canada; 5 Département de Microbiologie, Infectiologie et Immunologie, Université de Montréal 5622, Montreal, Quebec, Canada; 6 BIOQUAL, Inc. 10835 , Rockville, Maryland, USA; 7 Center for Human Systems Immunology, Duke University School of Medicine 12277, Durham, North Carolina, USA; 8 Department of Surgery, Duke University School of Medicine 12277, Durham, North Carolina, USA; 9 Department of Integrative Immunobiology, Duke University School of Medicine 12277, Durham, North Carolina, USA; 10 Duke Human Vaccine Institute, Duke University School of Medicine 12277, Durham, North Carolina, USA; Icahn School of Medicine at Mount Sinai, New York, New York, USA

**Keywords:** rhesus macaque, CD8+ T cell response, pox viral vector, next-generation liposomal adjuvant, ALFA, ALFQ adjuvants, SHIV challenge, immune response boosting, HIV-1 vaccine

## Abstract

To identify strategies for augmenting vaccine immunogenicity, we compared a pox-protein prime-boost regimen comprising recombinant modified vaccinia virus Ankara and multimeric HIV-1 Env gp145, adjuvanted with Army Liposomal Formulation (ALF) either adsorbed to aluminum salt (ALFA) or formulated with the QS-21 saponin (ALFQ), in rhesus macaques. ALFQ promoted greater magnitude and more durable humoral and cellular immune responses than ALFA, which exhibits similar immunogenicity to aluminum-based adjuvants. Peak Env-specific CD4^+^ T cell responses assessed by intracellular cytokine staining were 10-fold greater with ALFQ, and CD8^+^ T cell responses were unexpectedly robust, averaging greater than 1%. ALFQ induced higher levels of several immunostimulatory cytokines in plasma, which correlated with adaptive immune responses. However, vaccination did not protect against heterologous intrarectal challenge with transmitted/founder SHIV-CH505. We provide evidence that CH505 Env may maintain a relatively closed conformation, rendering it less susceptible to targeting by Fc-mediated antibody functions. Overall, ALFQ is a promising adjuvant to improve antibody and T cell response magnitude.

**IMPORTANCE:**

An effective and durable vaccine preventing HIV-1 acquisition is urgently needed to end the HIV-1 pandemic. To date, of the nine vaccine efficacy trials conducted in humans, only the RV144 vaccine trial demonstrated efficacy in reducing infections, although immune responses waned rapidly. We evaluated a modified HIV-1 vaccine regimen incorporating next-generation adjuvants to improve immune responses and vaccine efficacy in a gold standard, pre-clinical primate model. Adjuvanted protein boosting markedly increased antibody, T cell, and pro-inflammatory response magnitude. These data, combined with the adjuvant’s strong safety and immunogenicity track record in clinical trials, indicate that novel adjuvants represent a promising strategy for improving immune responses to protein immunogens. Future vaccine regimens against HIV-1 and other pathogens for which eliciting robust immunity has been difficult may benefit from incorporating these or related next-generation adjuvants.

## INTRODUCTION

An effective, durable, and accessible preventive strategy for HIV-1 is an urgent unmet public health need, with 1.3 million new HIV-1 infections in 2024 (1). To date, of the nine HIV-1 vaccine efficacy trials conducted in humans over the past 25 years, only the 2009 RV144 “Thai” trial demonstrated efficacy. The early VAX003 and VAX004 trials evaluating a gp120 protein immunogen failed to elicit the intended objective of neutralizing antibody responses (2, 3). While neutralizing antibodies remain a key goal for HIV-1 vaccine development, such responses have proven exceedingly difficult to achieve. Alternative vaccine strategies focusing on T cell responses using recombinant adenoviral serotype 5 (Ad5) vector also proved futile in the HVTN502 and HVTN503 trials (4, 5), as well as in HVTN 505, which incorporated a DNA prime (6). More recent trials (MOSAICO and IMBOKODO) aiming to elicit both T cell and antibody responses with an Ad26 mosaic prime and protein boost also failed to demonstrate protective efficacy (7), posing a major blow to the HIV-1 vaccine field with few remaining products in clinical development. In RV144, priming with ALVAC-HIV, a canarypox vector encoding *env*, *gag*, and *pol*, and boosting with AIDSVAX-B/E, a bivalent gp120 glycoprotein vaccine, achieved 60.5% efficacy at 12 months and 31.2% efficacy 3.5 years post-vaccination (8, 9), suggesting rapid waning of protective immune responses. Efficacy in this range was insufficient for product licensure. The follow-up HVTN702 trial conducted in South Africa sought to reproduce the RV144 outcome but was not efficacious (10). As a result, enthusiasm for pox-protein HIV-1 vaccination strategies is greatly diminished, although it has been argued that the numerous differences between the two trials limit the bearing of HVTN702 results on RV144 findings (11). There is now renewed emphasis on vaccines capable of eliciting neutralizing antibody responses, as well as acknowledgment that an effective vaccine will benefit from engaging multiple branches of immunity associated with lowering HIV-1 risk. Identification of strategies to enhance the magnitude, durability, and functionality of these vaccine-elicited adaptive immune responses has the potential to improve the effectiveness of future vaccines.

The partial efficacy of the RV144 trial allows assessment of immune responses associated with protection in a human vaccine trial. Correlates of reduced risk identified in the RV144 trial include HIV-1 envelope (Env)-specific IgG binding to the V1V2 region, low levels of Env-specific IgA in plasma, IgG3-mediated antibody-dependent cellular cytotoxicity (ADCC), antibody-dependent cellular phagocytosis (ADCP), and the magnitude and polyfunctionality of CD4^+^ T cells (12–14). IgG3 and ADCP were also associated with decreased risk of HIV-1 infection in the HVTN505 study, despite the lack of overall efficacy in this trial (15). Additional insight into protective immune responses has been gleaned from non-human primate challenge studies. Considerable evidence indicates that neutralizing antibodies against the Env protein mediate protection from HIV-1 acquisition, including correlations with reduced infection risk in NHP vaccine efficacy studies (16–21) and lower infection rates following passively administered neutralizing monoclonal antibody to individuals at high risk of HIV-1 infection who were exposed to sensitive strains (22). However, antibodies capable of neutralizing broad HIV-1 strains have yet to be elicited at sufficient titers in humans by vaccination. Other NHP trials have demonstrated similar immune correlates as RV144, with non-neutralizing binding antibodies (17, 23–26) and antibody effector functions such as ADCP, ADNP, and ADCC inversely correlating with infection risk (23, 26–31). These non-neutralizing humoral responses, alone or in combination with other adaptive immune responses correlated with reduced risk of infection, may be a more achievable vaccine goal than the elicitation of broadly neutralizing antibodies.

Adjuvants represent a powerful and understudied tool for modulating HIV-1 vaccine-induced immune responses. Most vaccines licensed for human use include aluminum salt (alum) as an adjuvant, achieving protective levels of immunity for numerous pathogens and backed by over 50 years of safety data. However, to overcome challenges in populations with impaired or senescent immune systems, as well as challenges stemming from weaker immunogens, alternative adjuvants have been developed and adopted in vaccines such as for herpes zoster, hepatitis B, and human papillomavirus. These adjuvants contain monophosphoryl lipid A (MPLA), a toll-like receptor 4 agonist, which activates dendritic and myeloid cells to induce an inflammatory response and increase immunogenicity (32, 33). In the case of the highly effective Shingrix shingles vaccine, further increases in immunogenicity were achieved through the incorporation of the saponin QS-21, which is contained in the AS01B adjuvant. The use of superior adjuvants that enhance vaccine responses represents an important consideration for the development of an effective HIV-1 vaccine.

We previously reported greater immunogenicity and efficacy of a pox-protein vaccine regimen adjuvanted with MPLA-containing Army Liposomal Formulation adsorbed to alum (ALFA) in rhesus macaques than with alum adjuvant alone (28). ALFA adjuvant reduced the per-exposure risk of SHIV acquisition by 90% in male macaques. Protection correlated with antibody effector functions ADCP and antibody-dependent neutrophil phagocytosis (ADNP), immune correlates that are supported by RV144 and other macaque HIV-1 vaccine efficacy trials (17, 26, 27, 29). Here, we build on this finding by evaluating pox-protein vaccination adjuvanted with Army Liposomal Formulation containing QS-21 (ALFQ) (34), a promising adjuvant with demonstrated efficacy and/or immunogenicity against multiple pathogens, including SARS-CoV-2 (35–39), *Campylobacter jejuni* (40), influenza (41), malaria (42), and HIV-1 (43), as well as two completed phase 1 studies in humans (38, 44). We compare immunogenicity and efficacy against SHIV challenge using either ALFQ or ALFA in macaques to identify adaptive and innate immune responses modulated by adjuvanted pox-protein vaccination.

## RESULTS

### Vaccination schedule

**Fig 1:**
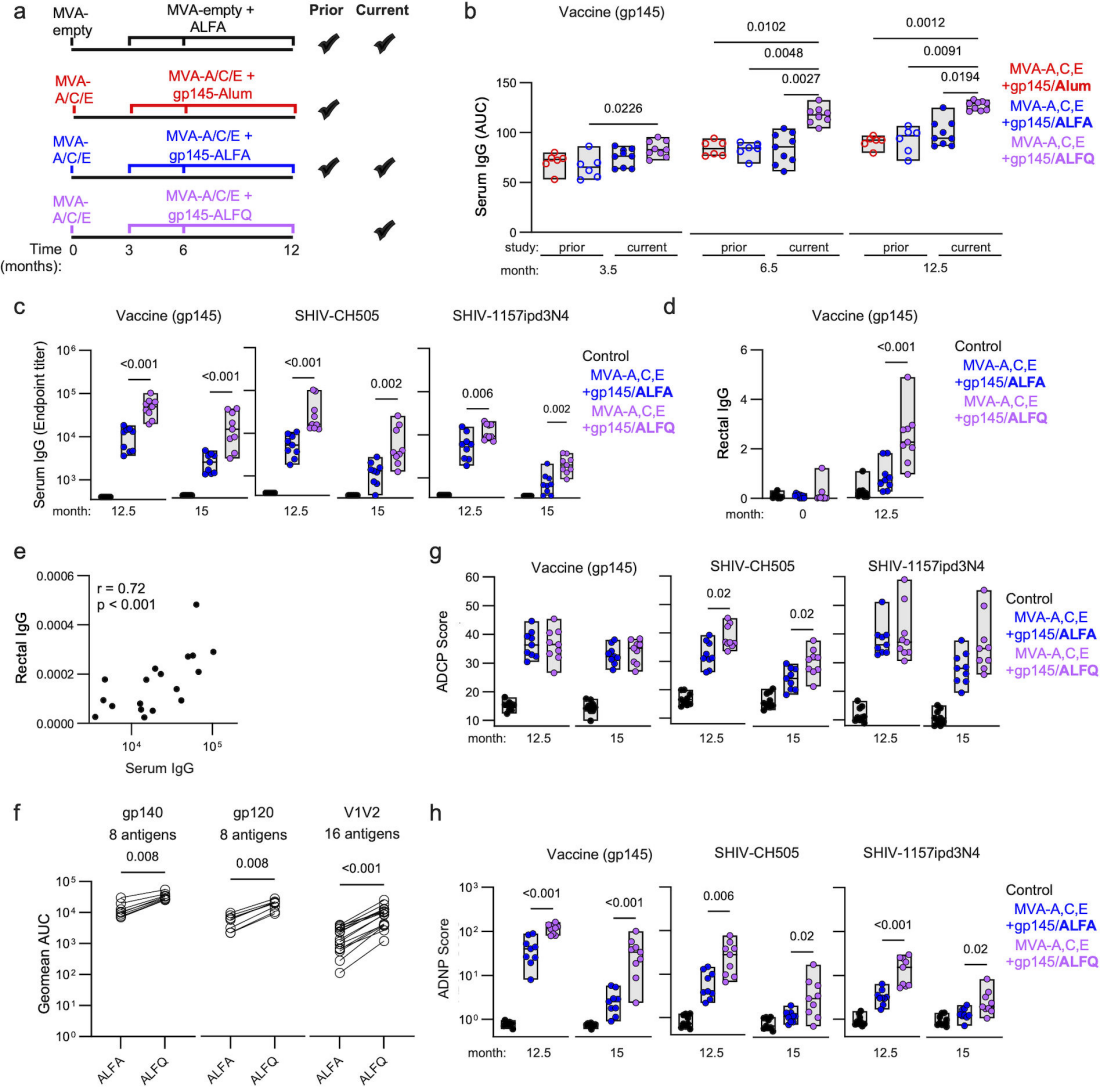
Study vaccination schedule and Env-specific antibody binding and effector responses elicited by vaccination**.** (**a**) Rhesus macaques were vaccinated at the indicated time points (arrowheads) with MVA or MVA and protein/adjuvant. (**b**) Serum IgG binding antibody responses to CO6980v0c22 multimeric gp145 are shown at indicated time points as the area under the curve (AUC) of ELISA binding curves. *P* values reflect Kruskal-Wallis test with Dunn’s multiple-comparison test. (**c**) Serum IgG binding antibody responses to CO6980v0c22 multimeric gp145, SHIV-CH505, and SHIV-1157ipd3N4 gp140 antigens are shown at peak (month 12.5) and 3 months after the last immunization (month 15). ELISA endpoint titer was calculated as the serum dilution giving a signal equal to two times the background value of the assay in wells not containing serum. (**d**) Rectal binding IgG responses at baseline and peak, measured as gp145 binding ELISA area under the curve/IgG concentration × 10^4^. (**e**) Non-parametric Spearman correlation between serum gp145 binding antibody titers at month 12.5 as shown in panel c and rectal binding IgG titers as shown in panel d. (**f**) Env-specific IgG responses to HIV-1 gp140, gp120, and gp70-scaffolded V1V2 antigens in serum at the time of challenge (month 15) were measured by BAMA. Paired comparison of group geometric mean AUC for each of the gp140, gp120, and V1V2 antigens. *P* values reflect paired Wilcoxon test. Serum ADCP (**g**) and ADNP (**h**) responses were assessed using THP-1 monocyte and human neutrophil effectors, respectively. Assays were performed with CO6980v0c22 multimeric gp145, SHIV-CH505, or SHIV-1157ipd3N4 gp140 antigens. Gray bars reflect range; black lines depict medians; *P* values in panels c, d, g, and h reflect the Mann-Whitney test comparing ALFA- with ALFQ-vaccinated animals. Symbol colors reflect control (black), alum (red), ALFA (blue), and ALFQ (purple) groups.

Twenty-seven male rhesus macaques were enrolled in three vaccine arms (*n* = 9 per group) in an HIV-1 vaccine immunogenicity and efficacy study involving a transmitted/founder (T/F) SHIV challenge. Vaccine priming consisted of a mixture of three live-attenuated recombinant modified vaccinia virus Ankara (MVA) vectors encoding HIV-1 *env*, *gag*, and *pol* from clades A, C, and CRF01_AE (45)*,* followed by boosting at months 3, 6, and 12 with the MVA mixture and a multimeric HIV-1 gp145 protein (CO6980v0c22, subtype C) (Fig. 1a) (46). This gp145 Env protein spontaneously forms multimers, with the trimeric form predominating in the enriched trimeric preparation used for the inoculum. The primary objective of our study was to elicit and evaluate the protective efficacy of Env-specific humoral responses, with the protein boosts intended to focus the immune response on HIV-1 Env. The vaccine design for this study was modeled after that of a previous study in which 90% vaccine efficacy was observed in male rhesus macaques when gp145 was adjuvanted with ALFA but not with alum (28). Here, gp145 was adjuvanted with either ALFA or ALFQ. Control animals received MVA constructs lacking HIV-1 inserts and ALFA adjuvant alone.

### Magnitude, breadth, and durability of binding antibody responses

To benchmark immune responses elicited between the current study and prior study (28), we measured binding antibody titers against the vaccine gp145 envelope by ELISA 2 weeks following each protein boost (Fig. 1b). At every time point, binding antibody titers elicited by ALFA were equivalent, with no statistically significant differences between the two ALFA groups or between the ALFA and alum groups. In contrast, the ALFQ adjuvant group mounted Env-binding antibody titers higher than those in the previous ALFA group at month 3.5 and higher than those in all ALFA and alum groups at months 6.5 and 12.5, with titers reaching peak levels in all groups at month 12.5.

Binding, neutralizing, and functional antibody responses were then assessed against an antigen panel comprising the vaccine gp145 and two additional clade C Env antigens in serum at peak immunogenicity (month 12.5, 2 weeks following the fourth immunization) and at a durability time point (month 15, 3 months following the fourth immunization). The heterologous SHIV-CH505 and SHIV-1157ipd3N4 (herein named CH505 and 1157) Env proteins were selected as the primary immunogenicity endpoints because they are both widely used in the field, demonstrate a Tier 2 resistance profile by neutralization assay (47–49), and one, SHIV-CH505, is a T/F Env (50). Serum IgG endpoint titers were ~5-fold greater with ALFQ compared to ALFA against the gp145 immunogen and CH505 gp140 and ~2.5-fold greater against 1157 gp140 (Fig. 1c). Three months following the last immunization, binding titers remained significantly higher with the ALFQ adjuvant for all three antigens, and all ALFQ-adjuvanted animals maintained responses to the heterologous antigens, which was not observed with ALFA. These data support elevated peak responses by ALFQ, conferring greater binding antibody response breadth and durability. Antibody responses to the vaccine gp145 antigen were also present in the rectal mucosa (Fig. 1d), with peak titers in the ALFQ group being 3-fold greater in magnitude than those in the ALFA group (*P* < 0.001). Rectal antibody gp145-binding responses correlated with those in serum (Fig. 1e), consistent with transudated IgG from circulation or FcRn-mediated IgG transcytosis as a source of mucosal antibodies (51–54).

To further interrogate binding antibody response breadth, animal sera were assessed for Env-specific IgG reactive to a panel of 32 antigens of diverse clades via binding antibody multiplex assay (BAMA) (Fig. S1). Binding antibody titers were significantly higher using ALFQ adjuvant for multiple gp140, gp120, and V1V2 antigens at both peak immunogenicity and 3 months after the vaccine series (Fig. 1f; Fig. S1b; *P* = 0.008, *P* = 0.008, and *P* < 0.001, respectively). The magnitude of increase was most apparent for the V1V2 antigens. Antibody responses against the clade C CH505 and 1157 Env proteins measured by BAMA using gp140, gp120, and V1V2 antigens mirrored the overall BAMA and ELISA results, with significantly greater magnitude responses achieved by ALFQ than ALFA at both peak and durability assessments (Fig. S2).

### Effector antibody responses

Fc-mediated antibody effector responses have been implicated in vaccine-mediated protection against infection in multiple pre-clinical HIV-1 vaccine studies. We measured serum ADCP and ADNP responses against the vaccine gp145, as well as CH505 and 1157 clade C gp140 antigens, to assess homologous and heterologous responses. These antigens are uncleaved, mostly trimeric Env proteins with a relatively open conformation (46), relevant for non-neutralizing humoral activity. ADCP responses were higher in animals that received ALFQ adjuvant compared to ALFA for CH505, but not the other antigens (Fig. 1g). ADNP responses were significantly greater with ALFQ for all three antigens, with titers 2- to 5-fold higher 2 weeks after the final immunization and 2- to 13-fold higher 3 months post-immunization (Fig. 1h). For the heterologous Env antigens, durable responses were limited with ALFA adjuvant (*P* > 0.05 relative to controls), while ALFQ promoted persistent ADNP to both CH505 and 1157 gp140. These heterologous ADNP effector antibody responses correlated with Env-specific binding IgG responses (gp145: *P* < 0.001, *r* = 0.96; CH505: *P* = 0.002, *r* = 0.69; 1157: *P* = 0.008, *r* = 0.60), suggesting that the adjuvant-mediated increase in effector antibody responses is likely achieved through increasing the overall magnitude of the binding antibody response. The ALFQ-adjuvanted vaccine therefore elicited ADCP and ADNP titers of comparable or greater magnitude than the ALFA-adjuvanted regimen, which previously achieved greater effector antibody responses than the alum-adjuvanted regimen using the same immunization regimen (28).

While neutralizing antibodies are generally not elicited by pox-protein vaccine regimens and were not the objective of this study, we evaluated neutralizing activity against the vaccine homologous and heterologous subtype C strains, CH505 and 1157, both with Tier 2 sensitivity. Vaccinated animals did not mount neutralizing antibody responses above the background levels seen in control animals (ID50 titers < 100; Fig. S3). Taken together, these studies demonstrate that adjuvanting pox-protein vaccination with a liposomal TLR-4 agonist plus QS-21 promotes greater magnitude binding, non-neutralizing effector antibodies with increased breadth than a similar adjuvant lacking QS-21.

### Substantial antigen-specific CD4^+^ and CD8^+^ T cell responses

**Fig 2:**
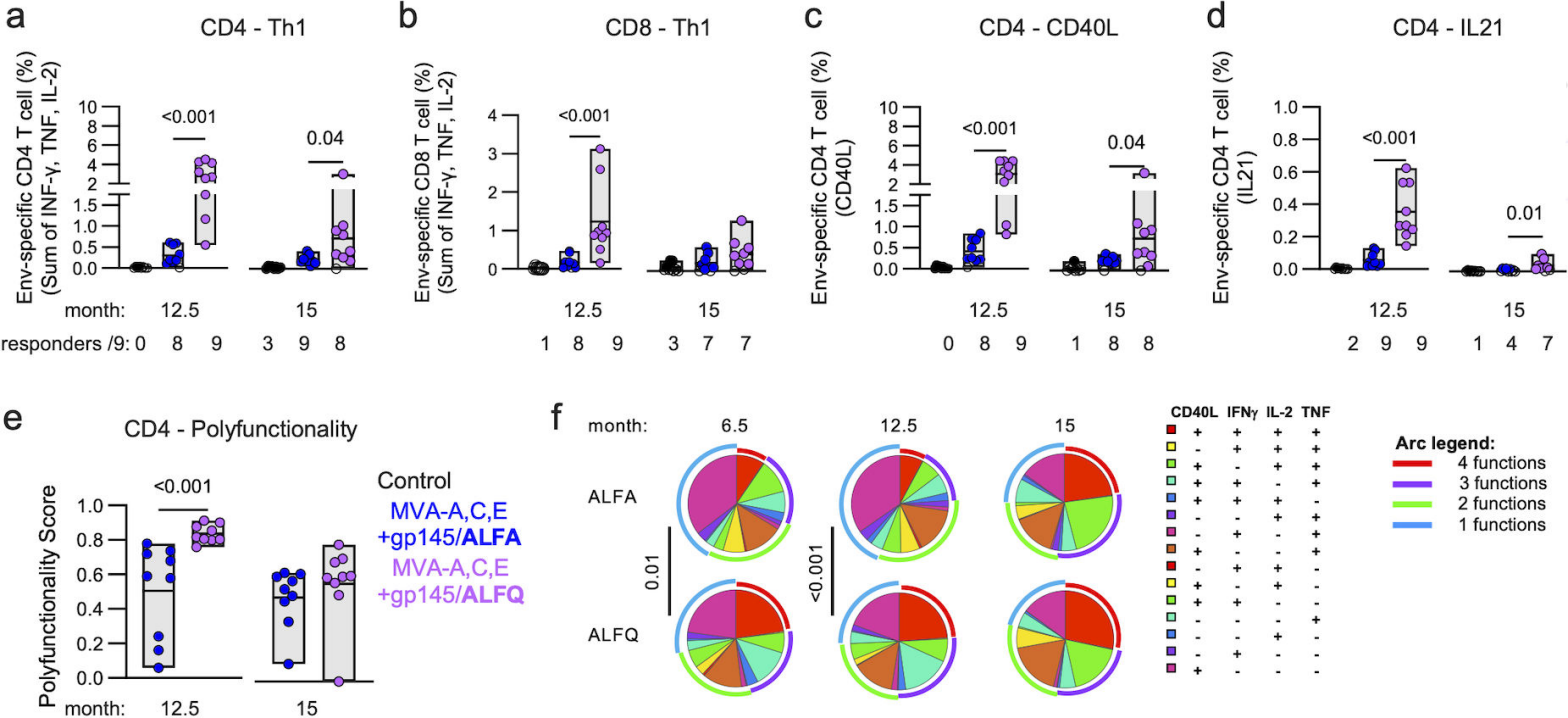
Antigen-specific CD4^+^ and CD8^+^ T cell responses. PBMC HIV-1 Env-specific CD4^+^ (**a**) and CD8 (**b**) T cells were measured by ICS for IFNγ, TNFα, and IL-2 2 weeks following the fourth immunization (month 12.5) and at the time of first challenge (month 15). Expression of CD40L (**c**) and IL-21 (**d**) was also measured on CD4^+^ T cells. The frequency of memory cells that expressed one or more cytokines following *ex vivo* Env PTE peptide pool stimulation is shown for each animal by arm (sum of two pools of 480 peptides total, Lot 150019). Boolean combinations of cytokine-positive memory T cell responses were summed in panels a and b. Positive responses, defined as greater than three times the group background at baseline, are depicted as closed symbols, and the positive rate within each group is indicated below each graph. (**e**) PBMC CD4^+^ T cell polyfunctionality score was assessed by COMPASS at months 12.5 and 15. Gray bars depict range; black lines depict means. *P* values reflect Mann-Whitney test comparing the two vaccine arms. Symbol colors reflect control (black), ALFA (blue), and ALFQ (purple) groups. (**f**) CD4^+^ T cell response functionality was assessed by Boolean cytokine combinations represented within the HIV-specific response using SPICE. Pie charts show the average fraction of the response comprised by each Boolean combination for each active arm. Predominant Boolean combinations are annotated in the pie charts for reference. *P* values reflect permutation test comparing the two vaccine arms for each time point.

HIV-1-specific T cells provide critical help for effective adaptive immune responses and mediate the killing of infected cells. To assess the impact of adjuvant on T cell responses, we measured antigen-specific T cells in peripheral blood mononuclear cells (PBMCs) by *in vitro* peptide stimulation and staining for intracellular expression of Th1 cytokines. All animals mounted Env-specific CD4^+^ T cell responses within 2 weeks following the second immunization (month 3.5), followed by maximal, consistent magnitude responses 2 weeks following the third and fourth immunizations (months 6.5 and 12.5) (Fig. S4). Peak CD4^+^ T cell responses in the ALFA group averaged 0.3% of the memory subset, consistent with levels previously reported for this regimen adjuvanted with either ALFA or alum (28) (Fig. 2a). In contrast, ALFQ group responses ranged from 0.5% to 4.5% (mean 2.9%), 10-fold greater than those observed with ALFA (*P* < 0.001).

Env-specific CD8^+^ T cell Th1 responses were also generated by the adjuvanted regimens. For ALFA, responses peaked at 0.03–0.5% (mean 0.2%), while those in the ALFQ group were several fold greater and ranged from 0.2 to 3.1% (mean 1.2%) (Fig. 2b). Peak Env-specific CD4^+^ and CD8^+^ T cell responses strongly correlated with one another (Spearman *r* = 0.72, *P* < 0.001) (Fig. S5), implicating similar or related mechanisms governing induction of adaptive T cell responses in the context of adjuvanted pox-protein vaccination.

To further characterize the functionality of Env-specific CD4^+^ T cells, two additional helper effector molecules were measured: CD40L, a surface protein essential for expansion of antigen-specific B cells, germinal center development, and CD8^+^ T cell responses (55); and IL-21, a surrogate marker of peripheral T follicular helper function. CD40L^+^ CD4^+^ T cell responses were robust and similar in magnitude to the Th1 cytokine responses (Fig. 2c). IL-21 was also expressed by a subset of CD4^+^ T cells elicited by ALFQ adjuvant (0.1–0.6%) (Fig. 2d). Both CD40L and IL-21 CD4^+^ T cell responses were significantly greater with ALFQ adjuvant than with ALFA at the peak response and 3 months after the last immunization.

Polyfunctional CD4^+^ T cell responses elicited by vaccination correlate with protection against infection with multiple pathogens, including HIV-1 in the RV144 clinical trial, suggesting that the simultaneous production of multiple cytokines aids in pathogen control (56, 57). Therefore, we assessed the quality of the CD4^+^ T cell response by Boolean combinations of Th1 cytokines and CD40L expressed by Env-specific cells. The ALFQ-adjuvanted vaccine induced highly polyfunctional Env-specific CD4^+^ T cells with a significantly higher COMPASS polyfunctionality score than the ALFA-adjuvanted vaccine at peak (Fig. 2e). For example, a greater proportion of the Env-specific cells expressed three or four functions with ALFQ adjuvant at months 6.5 and 12.5 (Fig. 2f). Three months post-last vaccination, response polyfunctionality was more comparable between study groups. We observed no evidence of Env-specific T cell responses in rectal mucosa (Fig. S6).

### Adaptive immune responses to MVA viral vector

Since strong CD8^+^ T cell responses have not previously been observed in studies using ALFQ-adjuvanted protein subunit vaccines (35, 58), we investigated potential mechanisms by which the MVA viral vector may have contributed to augmented CTL responses in the context of ALFQ. The recombinant MVA vectors encoding HIV-1 *gag*, *pol*, and *env* were administered contralaterally to the ALFQ adjuvant, raising the possibility of indirect systemic interactions between the viral vector and the adjuvanted protein immunizations. We measured MVA-specific immune responses in the form of binding antibodies to the vaccinia virus envelope protein by ELISA and T cell responses by ICS following MVA co-culture. Humoral responses to MVA increased modestly following the second immunization; however, no differences were apparent between study groups (Fig. S7a). CD4^+^ T cell responses to MVA were generally low and limited primarily to the control group (Fig. S7b), suggesting T cell responses in the active arms preferentially target the HIV-1 inserts over vector epitopes. To test this hypothesis, we measured Gag (transgene)-specific T cell responses. CD4^+^ T cell responses increased among most ALFA-adjuvanted animals following the second immunization (Fig. S7d), consistent with the T cell responses focusing on the vector inserts rather than the viral vector. Gag-specific CD4^+^ T cell responses to the boosting immunization were less apparent within the ALFQ adjuvant group. MVA-specific CD8^+^ T cells were observed in a subset of animals in each group but again did not differ between the ALFA and ALFQ arms (Fig. S7c). Taken together, while the MVA vector was immunogenic, eliciting vector-specific adaptive immune responses, the magnitude and quality of the vector-specific responses were not modulated by the adjuvanted protein immunization. Therefore, the remarkable Env-specific CTL responses were unlikely to be the result of ALFQ augmenting MVA immunogenicity.

### Innate immune activation profiles following immunization

**Fig 3:**
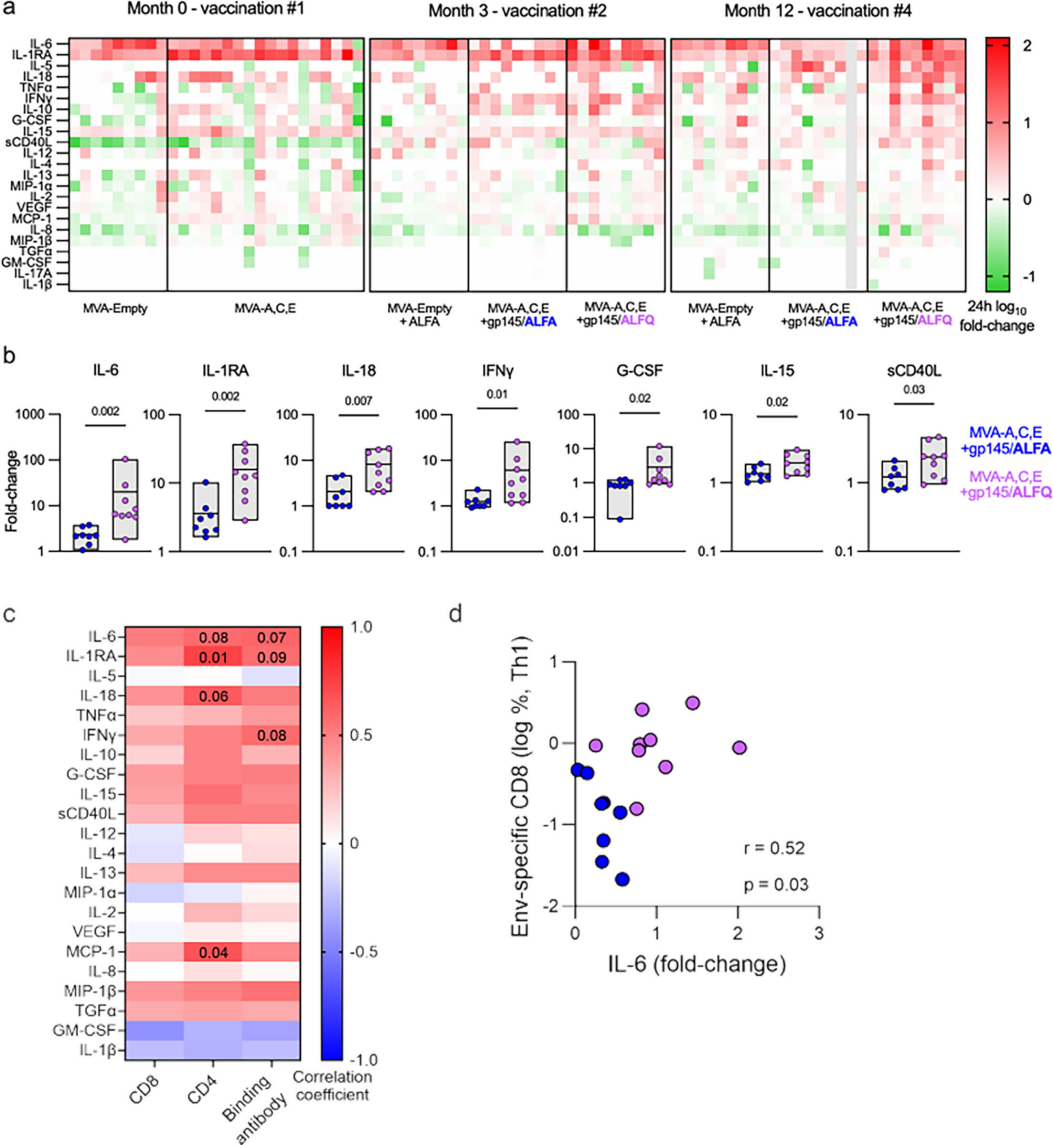
Plasma cytokines elicited by vaccination. Plasma cytokine elicitation expressed as fold change between the time of vaccination and 24 h following vaccinations 1, 2, and 4 (months 0, 3, and 12). (**a**) Heat maps display the log10 fold change in cytokine expression as measured by Luminex in each animal between the time of vaccination and the indicated time point. Each column represents an individual animal. Due to assay failure, no data were collected for one animal in the ALFA group at month 12.5. (**b**) Cytokines with significant differences in elicitation between the two active arms at month 12 are shown. Gray bars depict range; black lines depict means. *P* values reflect unadjusted Mann-Whitney tests. (**c**) Heat map depicts association between plasma cytokine levels (fold change in cytokine expression 24 h following the month 12 vaccination for ALFA and ALFQ groups) and peak adaptive immune responses at month 12.5: CD8^+^ and CD4^+^ Th1 cytokines (IFNγ, TNFα, and IL-2) and gp145 binding antibody endpoint titers. Spearman rho is displayed by heat map shading, and significant, FDR-adjusted *P* values are overlaid. (**d**) Bivariate plot depicting association between IL-6 induction 24 h following vaccination at month 12 and CD8 Th1 responses at month 12.5. Symbol colors reflect control (black), ALFA (blue), and ALFQ (purple) groups.

Immunostimulants contained within adjuvants trigger a rapid innate immune response characterized by the production of inflammatory cytokines and chemokines, which recruit and activate antigen-presenting cells responsible for generating robust adaptive immunity. To elucidate differences in innate immune activation elicited by ALFA and ALFQ, we measured levels of 23 cytokines/chemokines in plasma 1 day following the immunizations at months 0, 3, and 12 using multiplex Luminex assay. The greatest increases in cytokine expression were observed for IL-6 (geometric mean, 6.2-fold), a cytokine with both pro- and anti-inflammatory effects that can promote IgG secretion by B cells (59), and IL-1RA (geometric mean, 8.6-fold), which serves an anti-inflammatory role by inhibiting IL-1β-mediated inflammation (Fig. 3a) (60).

These cytokines were elevated by the initial MVA vaccination and were not specific to either ALFA or ALFQ adjuvants. Many cytokines were induced at equivalent levels following each vaccination, while other cytokines varied by vaccination. For example, among controls, IL-1RA induction was less pronounced following the fourth vaccination compared to the first, while sCD40L production was greater following the second and fourth immunizations (Fig. S8). ALFA-adjuvanted animals displayed a pattern of decreased cytokine induction over sequential immunizations, with significant reductions in IL-6, IL-1RA, IL-18, IL-15, and MCP-1, while IL-5 expression increased at month 12 and IFNγ expression increased at month 3. In contrast, cytokine expression generally increased following sequential ALFQ-adjuvanted boosting, with significant increases in IL-5, IL-18, IFNγ, and sCD40L following serial protein immunizations relative to the first MVA prime.

To further assess adjuvant-specific effects, we compared cytokine induction between ALFA and ALFQ adjuvants at months 3 and 12. Plasma cytokine responses were similar between the groups following the first protein boost, but greater with ALFQ by the third protein boost for seven cytokines (Fig. 3b). The strongest increases were in IL-6 (8.6-fold), IFNγ (4.9-fold), which is expressed by immune cells and directly inhibits viral replication along with additional immunostimulatory effects (61), IL-1RA (4.4-fold), and IL-18 (4.0-fold), a highly pro-inflammatory cytokine that facilitates type 1 T cell responses and induces IFNγ expression (62). Thus, MPLA-containing liposomal adjuvants administered in combination with MVA promote the production of inflammatory cytokines involved in adaptive immunity, and the addition of QS-21 facilitates recurrent inflammatory responses to repeated protein boosts.

### Plasma cytokine correlates of adaptive immune responses

We aimed to identify adjuvant-induced innate immune features that contribute to increased vaccine immunogenicity. Plasma cytokines induced by vaccination were evaluated for correlations with the magnitude of Env-specific T cell and antibody responses following the last immunization, applying FDR correction (Fig. 3c, *P* < 0.1). Both CD4^+^ Th1 and binding antibody responses positively correlated with post-vaccination increases in IL-6 and IL-1RA, the cytokines with the greatest fold increase in the ALFQ group. Additionally, IL-18 and MCP-1, a chemoattractant responsible for recruiting monocytes/macrophages and whose receptor is highly expressed on memory CD4^+^ T cells (63), correlated with CD4^+^ T cell responses. IFNγ correlated with binding antibody responses. To further investigate factors contributing to the unexpected CD8^+^ T cell responses, we assessed correlates without FDR correction as an exploratory analysis. The only plasma cytokine associated with CD8^+^ T cell response magnitude was IL-6 (Fig. 3d; *P* = 0.03), which has previously been associated with enhanced survival and proliferation of CD8^+^ T cells (64).

Given the multiple helper functions mediated by CD4^+^ T cells, we hypothesized that CD4^+^ T cells contributed to the development of both humoral and CTL responses. Indeed, Env-specific CD4^+^ T cell response magnitude correlated with Env-specific antibody and CD8^+^ T cell responses at both the peak and durability time points (Fig. S5). The strength of the correlation was highest 2 weeks following vaccination. To determine the factors driving high responses following adjuvanting specifically with ALFQ, we performed the same analyses using only the ALFQ group. Despite the reduced power with fewer animals, CD4^+^ Th1 responses remained correlated with binding antibody titers at peak immunogenicity (*r* = 0.75, *P* = 0.03). Taken together, these data implicate ALFQ-induced inflammatory cytokines in the augmentation of vaccine-elicited adaptive immune responses.

### Efficacy against mucosal transmitted founder SHIV challenge

**Fig 4:**
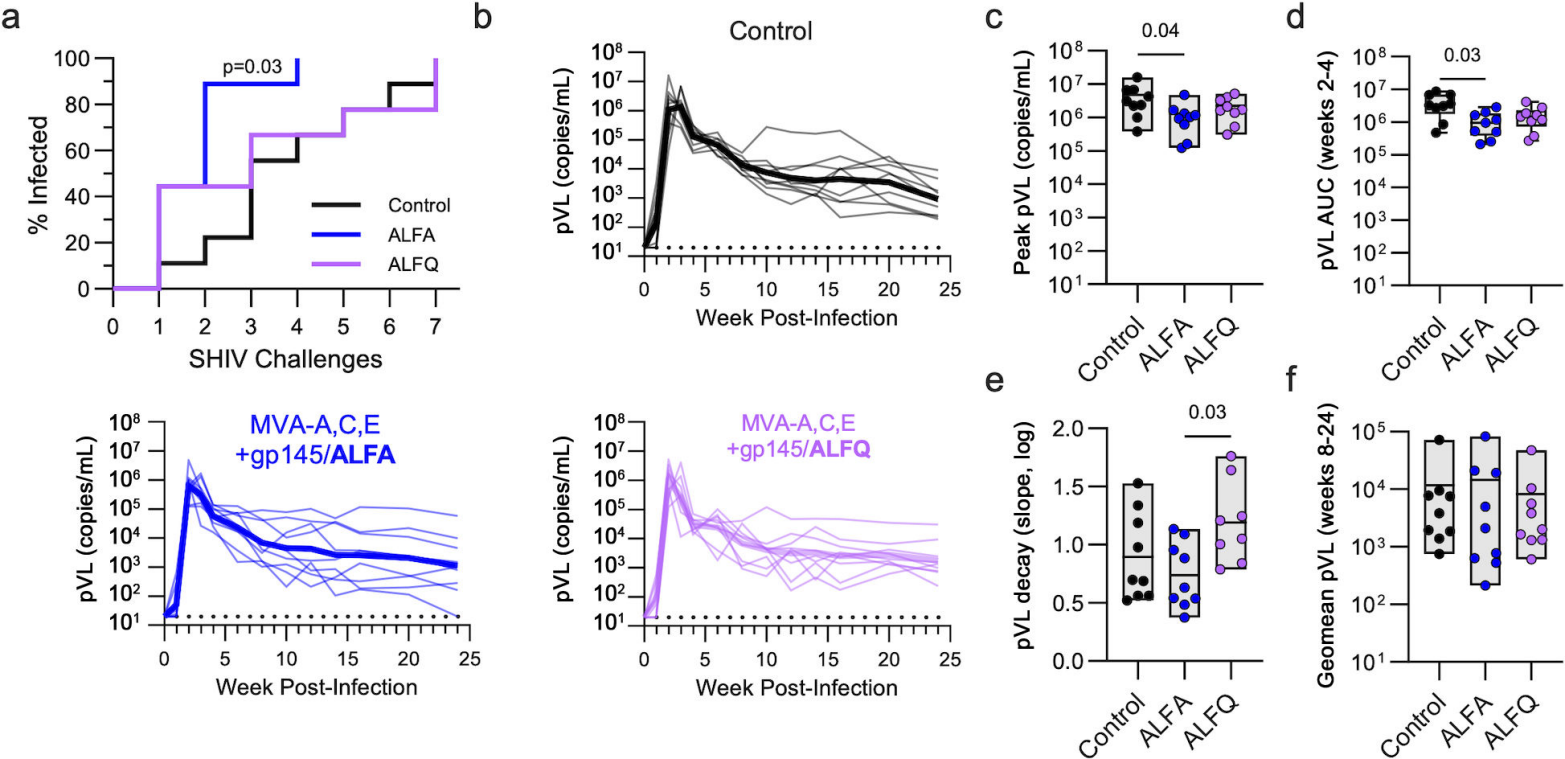
Vaccine efficacy against heterologous SHIV-CH505 challenge. (**a**) Kaplan-Meier curves depict SHIV infection acquisition following serial, every-other-week challenges. Differences between study groups were compared using Cox proportional hazards model. (**b**) Plasma viremia normalized by the time of infecting challenge is shown for each group. Thick line indicates the group geometric mean, and thin lines represent individual animals. Peak viral load (**c**), area under the curve (AUC) of early viremia (**d**), initial slope of decay from peak viremia to week 4 (**e**), and setpoint viral load (**f**) are shown by study group. Gray bars depict range; black lines depict means. *P* values reflect Kruskal-Wallis test with a Dunn’s posttest. Symbol colors reflect control (black), ALFA (blue), and ALFQ (purple) groups.

To determine whether vaccination conferred protection against infection, animals were challenged with a heterologous, difficult-to-neutralize SHIV. Serial limiting-dose intrarectal SHIV-CH505 challenges were administered every other week for up to 10 exposures per animal at an animal infectious dose of 40, beginning 3 months following the final immunization. SHIV-CH505 virus was selected as a stringent challenge strain for its transmitted/founder (T/F) Env, Tier 2 neutralization profile, and well-established viral replication kinetics and pathogenesis, which recapitulate human HIV-1 infection (47, 48). Time to infection was assessed by weekly plasma viremia measurements. The per-exposure infection rate was similar in control and ALFQ-adjuvanted animals (26% and 30%, respectively) and trended higher in the ALFA animals (56%, *P* = 0.03 relative to controls) (Fig. 4a). Vaccinated animals displayed modest differences in viral replication kinetics post-infection (Fig. 4b through f). Peak viral load, which occurred 2–3 weeks after the infecting challenge, as well as early viremia AUC, was significantly higher in control animals than in the ALFA active arm (Fig. 4c and d). Meanwhile, ALFQ-vaccinated animals displayed a more rapid viremia decay immediately following peak (Fig. 4e). Setpoint viral load did not differ between groups (Fig. 4f). These data indicate that while this regimen did not reduce infection risk against the T/F SHIV challenge, vaccine-elicited immune responses may modestly limit early viral replication.

As vaccinated animals appeared to suppress viremia faster than control animals, we assessed post-infection cellular immune responses for evidence of anamnestic responses among vaccinees. The control and active arms mounted comparable Env-specific CD4^+^ and CD8^+^ T cell responses 4 weeks post-infection (Fig. S9), rendering a robust anamnestic response unlikely. SIV Gag-specific CD8^+^ T cell responses were significantly lower in the vaccinated arms than in controls, potentially driven by the higher peak viremia antigen burden in control animals. This suggests that the HIV-1 *gag-pol* insert in the MVA vaccine did not prime an SIV Gag cross-reactive response. Vaccine-elicited Env-specific CD8^+^ T cell response magnitude (at either peak immunogenicity or at the time of challenge) did not correlate with measures of virus replication, including peak viremia, viremia 4 weeks post-infection, the slope of viremia decay, or plasma viremia AUC.

### Comparison of immunological and virological factors between adjuvanted vaccine efficacy studies

Discordant efficacy results between our prior challenge study with SHIV-1157 and the SHIV-CH505 virus used here, both of which used identical ALFA-adjuvanted MVA-gp145 vaccine regimens, prompted an investigation into immune and viral features that might explain the poor efficacy against the CH505 Env. As described above, serum antibody titers measured against the vaccine gp145 by ELISA within the same assay did not significantly differ between the two studies at the primary study endpoints post-vaccination (months 12.5 and 15; Fig. 1b). Furthermore, other humoral and T cell immunogenicity results were consistent between the two studies (28), indicating that the ALFA-adjuvanted regimen was similarly immunogenic in these two independent macaque cohorts.

**Fig 5:**
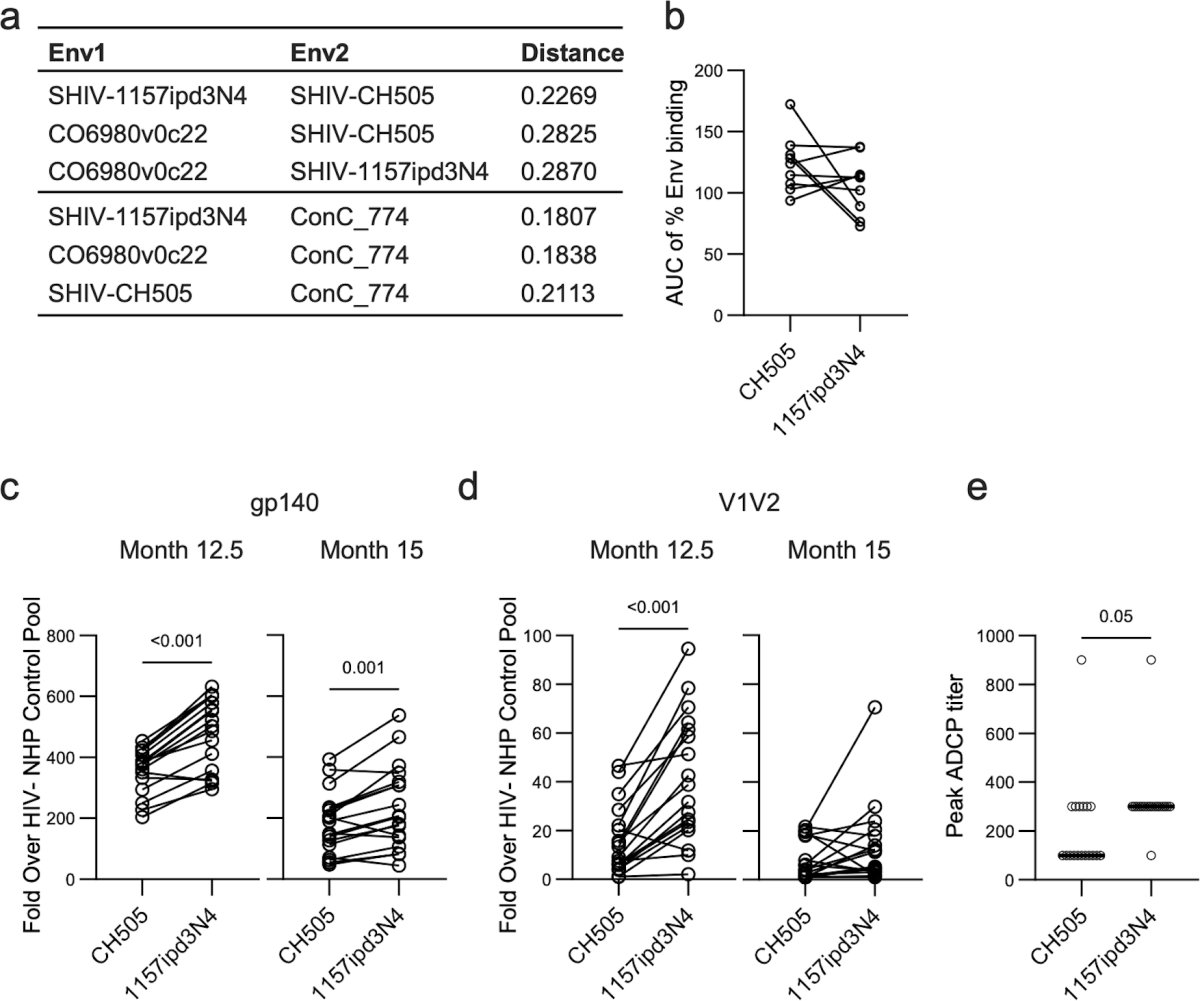
Comparison of ALFA-adjuvanted vaccine efficacy studies. (**a**) Genetic distance between the vaccine gp145 Env (CO6980v0c22) and the SHIV CH505 and 1157ipd3N4 Envs (top), as well as between these antigens and a consensus clade C Envelope sequence (ConC_774). (**b**) Competition ELISA was used to assess the ability of CH505 and 1157ipd3N4 gp140 proteins to block binding of sera (month 15) in ALFA-adjuvanted animals to gp145 antigen-coated plates. Paired Wilcoxon test, *P* > 0.05. (**c and d**) Antibody binding titers measured by Luminex assay in vaccinated animal plasma against the SHIV-CH505 and SHIV-1157ipd3N4 gp140 (**c**) or V1V2 (**d**) antigens. Data were normalized as fold change over pooled negative NHP sera. (**e**) ADCP responses among ALFA-adjuvanted animals measured against V1V2 antigens from CH505 and 1157ipd3N4. Peak ADCP responses serum reciprocal dilutions were compared using a paired Wilcoxon test.

We assessed potential differences in Env sequence that may impact the ability of this vaccine to protect against the two divergent SHIV strains. The 1157 and CH505 Envs have equivalent genetic distances from the CO6980v0c22 vaccine strain and the consensus C Env (ConC) (Fig. 5a; Fig. S10a). Since V1V2-specific responses have previously been associated with protection from HIV-1 infection (12), including in our SHIV-1157 challenge study, we also compared the V2 sequence similarity. Of the two challenge strains, the V2 region of CH505 is more genetically similar to the gp145 V2 (Fig. S10b), indicating that divergence in V2 sequence is likely not responsible for the lack of efficacy against SHIV-CH505.

We next considered that the Env-specific humoral responses elicited by this pox-protein vaccine regimen might target 1157 more efficiently than CH505. Antibody response reactivity against the different challenge Envs was measured by competition ELISA, comparing the ability of CH505 and 1157 gp140 proteins to compete with the binding of vaccinee sera to immobilized CO6980v0c22 gp145. The two challenge strain proteins both competed with gp145-binding antibodies to a similar extent (Fig. 5b), suggesting similar magnitude humoral responses to CH505 and 1157. We additionally used bead-based Luminex assays to measure sera antibodies to each antigen. Here, greater binding was observed to 1157 than to CH505 (for both gp140 and V1V2) (Fig. 5c and d), although potential differences in antigen conformation or immobilization may confound these results. A similar trend was observed when ADCP was measured against each challenge Env, with greater ADCP responses to 1157 gp140 and V1V2 (Fig. 5e; Fig. S11), suggesting that the CH505 V1V2 may be more resistant to the elicited ADCP. Taken together, these data implicate a possible role for limited binding antibody and ADCP responses against CH505 impacting efficacy in this challenge model.

Antibody effector functions, including the ADCP and ADNP immune correlates identified in our prior study, are mediated by antibody binding to Fc gamma receptors (FcγRs). FcγR allotypes influence their affinity for antibody binding (65). To assess whether differences in FcγR allotypes between the two studies influenced vaccine efficacy, we sequenced the *FcγR1, FcγR2A*, and *FcγR3A* alleles in control and ALFA animals enrolled in both studies. *FcγR1* and *FcγR3A* allotypes were largely equivalent between the two studies (Fig. S12a). *FcγR2A* frequency differed between the two studies, with heterologous 1/2 allotypes more common in Om et al. and homologous 1/1 allotypes more prevalent here. However, the time to infection for animals within each group expressing either the *FcγR2A* 1/1 or 1/2 allotypes did not differ from that of animals expressing other allotypes (Fig. S12b), suggesting that these allotypes were not linked to altered infection risk.

**Fig 6:**
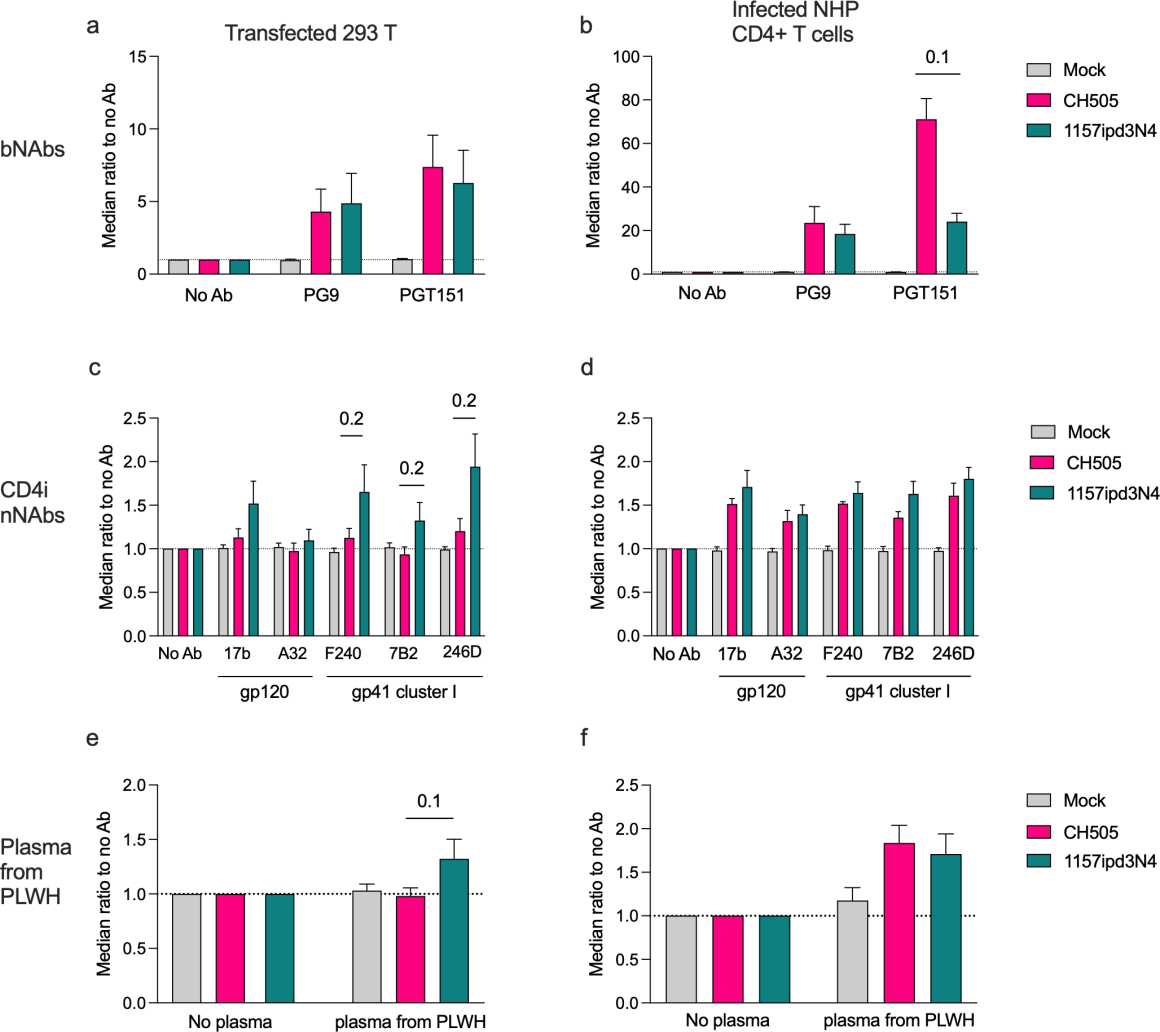
Conformation of challenge Envs on the cell surface. Binding of bNAbs (**a and b**), non-neutralizing antibodies (**c and d**), or plasma (**e and f**) indicated antibodies/plasma to CH505-375H or 1157 Env on the surface of (**a, c, e**) 293T cells transfected with Env-expression plasmids (*n* = 3), or (**b, d, f**) SHIV-infected rhesus macaque CD4^+^ T cells (*n* = 3), was measured by flow cytometry and reported as median ratio to no Ab. CD4i, CD4-induced antibodies; PLWH, people living with HIV. Dashed lines indicate relative staining level in the absence of added antibody/plasma. Bars indicate mean ± SEM. Significance was assessed between the two SHIV strains for each mAb using Mann-Whitney (*P* ≤ 0.2 are indicated).

The conformation of the HIV-1 envelope protein, whether it is in an “open” or more “closed” state, can influence susceptibility to antibody-mediated effector functions (66). To assess potential differences in the Env conformation for the two challenge strains, we expressed membrane-bound Env in two different cell types: 293T cells transfected with Env-expressing plasmids, and NHP CD4^+^ T cells infected with the two challenge viruses, followed by surface staining with a panel of broadly neutralizing (bNAbs) or non-neutralizing (nNAbs) monoclonal antibodies known to target distinct Env conformations. bNAbs PG9 (trimer apex-specific) (67) and PGT151 (gp120:gp41 interface-specific) (68) preferentially bind Env in a closed conformation, whereas the nNAbs bind Env following transition to an open, CD4-induced (CD4i) conformation. Here, PGT151 exhibited a trend toward greater binding to CH505 than 1157 in infected rhesus CD4^+^ T cells (Fig. 6b; *P* = 0.1), while PG9 binding did not differ between 1157 and CH505. Differential binding of bNAbs between the 293T and NHP cells is likely due to the absence of CD4 expression on the surface of 293T cells, with primary rhesus cells representing a potentially more physiological model for evaluating bNAb binding in the context of CD4. Among CD4i nNAbs, three showed trends of increased binding to 1157 relative to CH505 in 293T cells (Fig. 6c), suggesting that 1157 naturally adopts a more open conformation in the absence of CD4.

Plasma from people living with HIV, which tends to favor Env in an open conformation, also recognized 1157 modestly more than CH505 in 293T cells, although with less distinction in rhesus cells (Fig. 6e and f). Together, these data suggest that 1157 Env adopts a slightly more open conformation than the CH505, which may facilitate binding of non-neutralizing antibodies and render SHIV-1157 more susceptible to antibody-dependent effector functions.

## DISCUSSION

Here, we report the results of a pox-protein vaccination regimen in rhesus macaques in which the novel QS-21-containing liposomal adjuvant ALFQ promoted greater magnitude immune responses than the ALFA adjuvant, which lacks QS-21. Env-specific binding antibody responses were 5-fold greater with ALFQ adjuvanting. In addition, the breadth of the Env-specific antibody responses increased, with greater reactivity to antigens spanning multiple subtypes. T cell responses elicited by vaccination with ALFQ were particularly remarkable, with Env-specific CD4^+^ T cells comprising 1.3–6% and unexpected CD8^+^ T cell responses of 0.2–3.1%. The gains in humoral and cellular response magnitude were apparent after just two protein immunizations, were sustained following the third, and were also observed relative to the conventional aluminum salt adjuvant. Innate activation induced by ALFQ likely contributed to the augmented immunity following repeated immunizations. While both liposomal adjuvants promoted production of inflammatory cytokines involved in adaptive responses, immunostimulation was more sustained with ALFQ adjuvant over the course of repeated protein boosts. These data support ALFQ as a promising adjuvant to achieve efficient, augmented humoral and T cell immunity.

The ALFQ liposomes stimulate immune responses via multiple components, including the TLR4 agonist MPLA (69), and the saponin QS-21, which promotes development of a balanced Th1/Th2 response along with cytotoxic T lymphocyte production (70). Prior studies evaluating ALFQ-adjuvanted vaccines demonstrated strong adaptive immune responses in primates and humans (38, 44). Direct comparisons between ALFQ and other adjuvants have been limited to date. In the context of SARS-CoV-2 Spike vaccination adjuvanted with either aluminum hydroxide or ALFQ, ALFQ increased humoral and CD4^+^ T cell responses, but not CD8^+^ T cell responses (36). In the context of an HIV-1 Env protein vaccination, ALFQ enhanced peak binding antibody titers compared to alum (58); however, this enhancement was not sustained 3 months post-vaccination, suggesting that the addition of the pox vector prime used here may enhance the durability of ALFQ-elicited antibody titers. In addition, we observed heightened humoral responses across a large panel of HIV-1 isolate gp120, gp140, and V1V2 antigens with ALFQ compared to ALFA (and, by extension, to alum [28]). V1V2-specific responses, in particular, may benefit from the ALFQ adjuvant, although a similar difference was not observed by Saunders et al. (58). Further study using matched Env isolates across the antigen panels and evaluating the contribution of base-specific antibodies to the binding responses will aid in determining whether adjuvants influence humoral response epitope specificity. In our previous study using ALFA adjuvant and the same vaccine regimen used here, immune responses were comparable between ALFA and alum for most immune functions, with the exception of improved antibody-dependent phagocytic responses with ALFA (28). Our study offered an opportunity to assess whether potent adjuvants interfere with immunogenicity of concurrently delivered viral vector immunizations. We did not see strong evidence of adjuvanted protein vaccination altering T cell responses to the HIV-1 transgenes. CD4^+^ T cell responses to the pox vector were quite limited when adjuvanted protein was also administered. Future HIV-1 vaccine development may benefit from further study of potential interactions between the co-delivery of multiple vaccine platforms.

Robust antigen-specific CD8^+^ T cell responses from protein vaccination were surprising, as protein vaccinations typically yield low-magnitude CD8^+^ T cell responses due to exogenous antigen presentation on MHC class II, even when using the ALFQ adjuvant (35). Higher magnitude responses are generated by some viral vectors and highly optimized T cell epitope vaccines (71–74). Mechanisms underlying CD8^+^ T cell responses to adjuvanted protein are not well understood, but antigen cross-presentation has been observed for other saponin- and MPL-based adjuvants, such as AS01 and ISCOMATRIX (75, 76). Previous work with QS-21 demonstrated its localization within CD169^+^ resident macrophages in the draining lymph node, where it recruits and contributes to dendritic cell phenotypic maturation, leading to increased CD8^+^ T cell responses (77). Saponins are also known to increase cytosolic translocation of antigen required for cross-presentation (78). TLR4 agonists, including MPLA, increase cross-presentation by increasing MHC class I trafficking into phagosomes (79) and reducing antigen degradation within phagosomes (80). MPLA and QS-21 have demonstrated synergy in mice for the development of strong adaptive immune responses, including CD8^+^ T cells (81). We identified the cytokine IL-6 as being modestly associated with CD8^+^ T cell responses, consistent with its role in enhancing the survival and proliferation of CD8^+^ T cells (64).

Despite these known adjuvant effects, the compelling CD8^+^ T cell immunogenicity observed here contrasts with previous studies using ALFQ or similar adjuvants in non-human primates and humans. Strong CD8^+^ T cell responses were not observed following ALFQ-adjuvanted protein nanoparticle vaccination against SARS-CoV-2 in rhesus macaques or humans (35–38), or against HIV-1 Env in a previous study using the similar AS01B adjuvant with a nanoparticle in humans (82). An additional study in rhesus macaques combining a DNA prime with ALFQ-adjuvanted protein boost also did not detect CD8^+^ T cell responses (43). One exception, however, is another pox-protein vaccine regimen, in which ALVAC priming followed by ALVAC plus ALFQ-adjuvanted Env protein boosting also elicited strong CD8^+^ T cell responses in NHPs (83). Taken together, these findings suggest that the combination of pox vector and QS-21-containing adjuvant is required for the observed CD8^+^ T cell response development. The MPLA within the liposomes may also contribute, although this was not evaluated. It is unlikely that ALFQ adjuvanted the MVA vaccine, as T cell responses to the MVA vector itself or the HIV-1 Gag insert did not differ between adjuvants. Another difference between these ALFQ-adjuvanted vaccine studies is the use of a soluble Env protein here (83) versus antigen display on a nanoparticle in the previous ALFQ studies. Nanoparticles themselves have been associated with induction of cross-presentation (84); however, the effect of combining multiple immunomodulatory strategies is unclear, and this cross-presentation effect may be lost when the nanoparticle is combined with the ALFQ liposomes. The longer 3- to 6-month intervals between vaccinations used here may also be important to achieve CD8^+^ T cell responses. One hypothesis we favor is that the viral vector provides additional immunostimulatory signals that complement QS-21, such as from dying pox-infected cells releasing “danger” signals that promote cross-presentation by antigen-presenting cells (85). Further work investigating the mechanism responsible for enhanced CD8^+^ T cell responses may aid in developing strategies that achieve consistent and robust CTL immunity from protein immunizations, a long-standing challenge in vaccinology. Next-generation T cell vaccine design may benefit from combining ALFQ-like adjuvanted pox-protein regimens with platforms that promote cytotoxicity against infected cells, such as live replicating vectors (86), to achieve high-magnitude responses with effective antiviral activity. Targeting Gag rather than Env, as discussed below, is likely also important.

The increased expression of many cytokines and distinct innate immune signatures early following ALFQ-adjuvanted vaccination likely supports the development of robust adaptive immune responses. Previously, early innate immune signatures have been shown to correlate with immune responses that reduce infection risk following pox-protein vaccination in humans (87). We found IFNγ, IL-6, and IL-1RA levels to be associated with subsequent binding antibody responses. IFNγ can induce class-switching in B cells (61), and increased IL-6 may promote IgG secretion (59). The association between IL-18 and CD4^+^ T cell response magnitude is consistent with IL-18 involvement in Th1 response induction (62). Maintenance of the early innate immune response to boosting vaccinations differed between the ALFA and ALFQ groups. Innate responses following ALFA-vaccination decreased with subsequent immunizations, suggesting desensitization to repeated ALFA exposure. In contrast, cytokine elicitation increased following repeated ALFQ-adjuvanted vaccinations. This could indicate a recall adaptive immune response or altered response kinetics upon subsequent exposure (e.g., IFNγ production by rapidly responding memory T cells). These early cytokine signatures may be valuable surrogates for development of robust adaptive immune responses. Taken together, these data suggest that QS-21-containing adjuvants may promote innate signatures that improve immunogenicity of repeated protein vaccinations.

Despite its potent immunogenicity, the vaccine did not protect against heterologous SHIV challenge or substantially reduce post-infection viremia in this challenge model. High-magnitude Env-specific binding antibody responses in both the serum and rectal mucosa, which would be expected to contribute to reduced risk of infection against rectal SHIV challenges (88), were not sufficient for vaccine efficacy. Using an identical vaccine regimen with the ALFA adjuvant, we previously demonstrated 90% efficacy in male rhesus macaques against heterologous challenge with a different SHIV, the 1157ipd3N4 clone, which does not contain a T/F Env (28, 49). The explanation for these discordant challenge results is unclear. Immune responses elicited in the two studies were of comparable magnitude. Genetic comparison of the challenge strain *env* sequences also indicated a similar genetic distance to the vaccine strain, including in the V2 region, which was previously identified as a target of protective antibody responses (12). Comparison of ADCP and binding antibodies against the two challenge Envs suggested lower cross-reactive titers against the CH505 Env used in this study than against 1157ipd3N4, despite equivalent vaccine-specific binding antibody responses, suggesting that the T/F CH505 may be less susceptible to protective immune responses elicited by this regimen. Of note, it was recently suggested that ADCP using recombinant Env-coated beads might not recapitulate ADCP responses against infectious viral particles (89), and that other assays for characterizing effector immune responses may be illuminating. One caveat of this analysis is the difficulty in normalizing results between antigens, and a different binding assay (competition ELISA) did not demonstrate a difference in antibody responses to the two challenge Envs; therefore, caution is warranted in interpreting these data. In addition, IgG binding and Fc effector functions were assessed using uncleaved trimeric proteins that might not display an authentic native Env conformation from the SHIV challenge virus. Proteins that adopt more closed conformations, the state assumed by most primary HIV-1 isolates, may better detect antibody responses predictive of protection against T/F viruses. Conformational analyses of the two challenge Envs using neutralizing and non-neutralizing monoclonal antibody binding profiles suggested that 1157ipd3N4 is slightly more open than CH505; confirmation of these results with additional bNAbs in future studies would be informative. Env “openness” has previously been associated with susceptibility to ADCC (66), lending support to the hypothesis that increased sensitivity of 1157ipd3N4 to effector antibody responses contributed to vaccine efficacy against SHIV-1157ipd3N4 but not SHIV-CH505. Future vaccination efforts seeking to promote non-neutralizing humoral responses to natural Env conformations may benefit from the use of immunogens that elicit greater cross-reactive V2-specific responses. Boosting immunogens that adopt more closed conformations may also aid in shepherding non-neutralizing responses towards slightly more native states.

Another possibility is that deleterious immune responses were elicited. For example, strong Th1 CD4^+^ T cell responses in blood were previously reported to correlate with reduced vaccine efficacy (90). However, robust and comparable CD4^+^ T cell immunity was achieved in the ALFA arms of the two challenge studies, making this explanation unlikely. Env-specific CD8^+^ T cell responses have been associated with increased HIV-1 viremia in humans, in contrast to Gag-specific responses, which have been associated with lowering viremia (91). Thus, high-magnitude Env-specific CD8^+^ T cell responses generated by ALFQ adjuvant may have increased infection risk, potentially negating protective effects of humoral antiviral activity. In this study, the ALFA-adjuvanted group appeared to have a higher per-exposure infection risk than the control group. We previously reported ALFA-dependent priming of mucosal HIV-1-specific CD4^+^ T cells with this vaccine regimen (28). Increased Env-specific CD4^+^ T cells in the mucosa, as well as stimulation of these cells upon SHIV challenge, may have increased infection risk in the absence of protective effector antibodies (92, 93). Future studies evaluating mucosal CD4^+^ T cell responses after ALFQ adjuvanting are necessary to determine whether QS-21-containing MPLA liposomal adjuvants also promote mucosal CD4^+^ T cell responses.

Vaccine efficacy against acquisition of a heterologous virus in NHP models is rare, and few studies use challenge strains encoding a T/F HIV-1 Env, which represents a potentially even higher bar. T/F Envs exhibit distinct characteristics relative to non-T/F Envs that aid their ability to infect target cells. T/F viruses have greater Env content per virion, cell-free infectivity, and IFNα resistance (94). It is possible that the CH505 T/F Env was more difficult to protect against than the 1157ipd3N4 Env used in our prior challenge. NHP challenge studies that previously achieved protection against T/F SHIV strains typically used homologous Env vaccine immunogens. For example, efficacy against SHIV-CH505 has been observed using CH505-lineage immunogens in a DNA/protein co-immunization (23) and with a stabilized CH505 Env trimer co-administered with a TLR7/8 agonist (95). Similarly, efficacy against SHIV-BG505, the other T/F SHIV used in multiple challenge studies, was evaluated following vaccination with homologous Env protein plus viral-vectored insert (19) or stabilized BG505 Env trimers (20). However, a recent study using computationally selected multivalent gp120 boost immunogens optimized for V1V2 binding antibody breadth is, to our knowledge, the first to report reduced infection risk against a heterologous T/F SHIV challenge (96). The long interval between boosting immunizations may have improved the quality of the humoral responses contributing to protection. Prior successes, limited largely to homologous T/F Env vaccine regimens, suggest that these strains may be more resistant to cross-reactive vaccine-elicited immune responses.

We demonstrate here the rapid, robust, and durable humoral and T cell responses achieved by a next-generation adjuvant, ALFQ, in the context of pox-protein vaccination. Antibody responses were broadly reactive against multiple HIV-1 subtypes and isolates. High-magnitude responses are consistent with other pre-clinical ALFQ-adjuvanted vaccine studies in primates and humans (35–37, 40–44), highlighting the potential clinical relevance of ALFQ for vaccines against diverse infectious diseases. Unique to this vaccine regimen was the unexpected, high-magnitude CD8^+^ T cell response, likely elicited via cross-presentation mediated by the combination of saponin, TLR4 agonist, and pox viral vector components. The elicitation of CD8^+^ T cell immunity in conjunction with high-titer humoral responses is a valuable response profile for multiple pathogens with unmet needs, including prophylactic and therapeutic vaccines targeting HIV-1.

## MATERIALS AND METHODS

### Animals

Twenty-seven colony-bred Indian-origin male 3- to 6-year-old rhesus macaques (*Macaca mulatta*) were assigned to three groups (nine animals per group) in a procedure resembling minimization. Group assignments were made deterministically by sorting all animals by age, pre-existing CN54 gp140 ELISA endpoint titer, and pre-existing (cross-reactive) p27 ELISA titer and then assigning them to groups 1, 2, and 3 in order. Additional adjustments were made to further balance the groups across these factors. Animals expressing protective *Mamu* alleles A*01, B*08, and B*17 were excluded from the study. The sample size was chosen to achieve at least 80% power to detect 80% vaccine efficacy in an individual vaccine arm, using a two-sided log-rank test with 5% type I error.

### Vaccine components

All vaccine components and procedures were identical to that of the previously described study (28), with the exception of the addition of the ALFQ adjuvant. Control animals received live-attenuated MVA lacking insert; active arm animals received an equal mixture of three recombinant MVA vaccines expressing HIV-1 *env* and *gag*/*pol* genes (45). The three MVA vaccines (MVA-A/C/E) were as follows: (i) MVA-CMDR, CM235 *env*, and CM240 *gag*/*pol* (subtype CRF01_AE); (ii) MVA-KEA, KNH1144 *env* and KER2008 *gag*/*pol* (subtype A); and (iii) MVA-TZC, TZA125 *env*, and TZA246 *gag/pol* (subtype C). Subtype C gp145 recombinant protein (CO6980v0c22) was expressed in CHO cells and enriched as a trimeric preparation containing ~60% physical trimers (46). For ALFA-adjuvanted vaccination, gp145 was adsorbed to aluminum hydroxide (Alhydrogel, Brenntag) at 300 µg aluminum per 0.5 mL dose. ALF liposomes consisted of 1.145 mM phospholipids (DMPC:DMPG ratio = 9:1), 43% cholesterol, and 100 µg of synthetic monophosphoryl 3-deacyl lipid A (3D-PHAD; MPLA:phospholipid ratio = 1:8.8; Avanti Polar Lipids) per dose in PBS. The gp145-Alhydrogel suspension was used to reconstitute microfluidized and lyophilized small unilamellar ALF to produce ALFA (97). ALFQ was prepared as previously described (97, 98). ALFQ liposomes consisted of 11.45 mM phospholipids (DMPC:DMPG = 9:1), 55% cholesterol, 100 µg 3D-PHAD (MPLA:PL = 1:88), and 50 μg QS-21 per dose in PBS. The resulting gp145 immunogen remains a soluble protein mixed with the liposomal adjuvant, though not displayed on its surface.

### Immunizations and challenge

Animals received an MVA priming immunization containing 10^8^ PFU of either empty-vector MVA or MVA with the HIV-1 inserts in 1.8 mL injections. Three boosting immunizations consisted of MVA plus the adjuvanted, multimeric subtype C gp145 protein administered contralaterally at 100 µg/dose in 0.5 mL at months 3, 6, and 12 following the prime. All vaccinations were administered intramuscularly, alternating between right and left quadriceps for each product at each vaccination. At month 15, all animals were serially challenged intrarectally with a 1:250 dilution of R5-tropic SHIV.C.CH505.375H.dCT (48), kindly provided by George Shaw, until they became viremic or for up to 10 prespecified challenges, with challenges spaced 2 weeks apart. This dilution represents the intrarectal animal infectious dose that results in 40% infection (AID40) for this stock, as determined by prior challenge of naïve rhesus macaques (B.K. Felber, personal communication).

### Binding antibody responses

#### Env-specific IgG ELISA

Sera were assayed in triplicate for the presence of Env-specific IgG antibodies by ELISA as previously described (99) except that horseradish peroxidase-labeled affinity-purified goat anti-monkey IgG (Alpha Diagnostics, Cat. #70021, polyclonal) was used as the secondary antibody. Briefly, 96-well Immulon 2 “U”-bottom microtiter plates (Thermo Scientific) were coated with 0.1 µg/well of CO6980v0c22 gp145, CH505.375H gp140, or 1157ipd3N4 gp140 protein for 2 h at 37°C and then blocked overnight at 4°C with 0.5% casein and 0.5% BSA in phosphate-buffered saline (PBS). Plates were washed, incubated for 1 h at 25°C with serial dilutions of test serum, washed, incubated with goat anti-monkey IgG (described above) for 1 h at 25°C, and washed again. ABTS substrate (KPL) was added for 1 h at 25°C. Plates were analyzed at 405 nm on a VersaMax Microplate Reader using the SoftMax Pro 7.1.0 software (Molecular Devices). Matched pre-immune sera were used as the negative control for each animal. Data are expressed as endpoint titers, calculated by interpolating from a four-parameter sigmoidal curve the dilution that yielded an optical density equal to twice the background value.

Rectal mucosal secretions were collected using Weck-Cel Swabs (BVI Medical) and stored at −80°C until use. Antibodies were eluted as described previously (100). Briefly, swabs were placed into Spin-X columns (Sigma), eluted twice with 300 µL cold elution buffer (1× Protease Inhibitor Cocktail Set I [Sigma], 0.25% BSA, PBS), and then filtered through a 0.22 µM membrane. Total IgG in eluted samples was measured using Monkey IgG ELISA Kit (Alpha Diagnostic, Cat. #7050), according to the manufacturer’s instructions. Env (gp145)-specific IgG was measured by ELISA. Briefly, U-bottom ELISA plates were coated with 0.1 µg/well of gp145 protein in PBS overnight at 4°C. Wells were then blocked for 1 h at 37°C with 5% skim milk powder and 0.05% Tween-20 in PBS at 4°C, washed, and incubated for 1 h at 37°C with serial dilutions of test sample. Plates were washed, incubated with mouse anti-monkey IgG (SB108A, Southern Biotech) for 1 h at 37°C, and washed again, followed by addition of 1-Step Ultra TMB ELISA Substrate (100 µL/well) (Thermo Scientific, Cat. #34029) for 10 min at 25°C. The reaction was stopped with the addition of 100 µL/well 1 N HCl. Plates were analyzed at 450 nm on a VersaMax Microplate Reader using the SoftMax Pro 7.1.0 software (Molecular Devices).

#### Competition ELISA

ELISAs were performed as outlined above for the rectal samples, except for the primary antibody conditions. For this step, a serial dilution of each of the two gp140 SHIV envelope proteins (CH505.375H and 1157ipd3N4 gp140) was performed in block buffer with concentrations spanning 100 μg/mL to 0.195 μg/mL gp140. Each of these protein dilutions was then mixed in a 1:1 ratio with 1:200 diluted serum samples, resulting in a final serum dilution of 1:400. Additionally, a positive control was prepared for each serum sample, consisting of the same 1:200 serum dilution mixed at a 1:1 ratio with block buffer. One hundred microliters of the mixture was then added in duplicate into microtiter plates alongside positive controls (1:400 serum without gp140) and a block buffer-only negative control and incubated for 1 h at 37°C. The percentage of the maximum binding to CO6980v0c22 gp145 for each challenge Env was expressed as the area under the curve of this measure after log transformation of the x-axis (serum dilution). A paired significance test (Wilcoxon) was used to test for differences in competition by the two SHIV strain gp140s.

#### MVA-specific antibody responses

ELISAs were performed to measure antibodies against the MVA viral vector as described above (see “Rectal mucosa” section), except that plates were coated with recombinant vaccinia virus cell surface-binding protein (D8L) (CusaBio, Cat. #CSB-EP322653VAA). Rabbit anti-D8L monoclonal antibody (CusaBio, Cat. #CSB-PA322653LA01VAA) was used as a positive control in a 2-fold serial dilution, beginning at 1:300,000. Binding was detected using donkey anti-rabbit IgG (H + L) (Jackson ImmunoResearch, Cat. #711-035-152).

#### Binding antibody multiplex assay (BAMA)

Binding antibody multiplex assay (BAMA) was performed blinded to vaccine arm as previously described (101) against a panel of globally diverse gp140, gp120, and V1V2 antigens (102). Briefly, carboxylated magnetic beads (Luminex Corporation) were covalently coupled to HIV-1 antigens and incubated with serially diluted serum samples (1:80, 5-fold, six dilutions). IgG was detected with goat anti-monkey IgG-biotin (Rockland) and PE Streptavidin (BD Pharmingen), followed by reading on a Bio-Plex 200 instrument (Bio-Rad). Assay positive controls included polyclonal IgG from people living with HIV-1 (HIVIG) and the HIV-1 mAbs CH31, CH58, and 2G12. Negative controls included HIV-1-seronegative human sera, blank (uncoupled), and murine leukemia virus (MuLV) (empty) gp70 scaffold-conjugated beads. Antibody titers were reported as area under the curve (AUC), calculated using the trapezoidal method from background- (wells containing antigen-coated beads with no sample) and blank-subtracted (uncoupled beads or MuLV [empty] gp70 scaffold beads) net median fluorescence intensity (MFI) values measured at each dilution. A response was considered positive if the net MFI values were (i) greater than 100, (ii) greater than or equal to the antigen-specific cutoff (95^th^ percentile of all baseline sample binding per antigen after high baseline exclusion), and (iii) greater than 3-fold of the matched baseline sample binding before and after blank subtraction.

#### Luminex binding antibody measurements

Uncleaved gp140 proteins were designed with R-to-S mutations in the gp120/gp41 furin cleavage site and incorporated the native leader peptide, full MPER sequence, followed by a short GGGS linker sequence, and a C-terminal AviTag. V1V2-gp70 scaffolds were designed as previously reported (103). Sequences, codon-optimized for expression in human cells, were synthesized (Genscript, Piscataway, NJ) and cloned into a custom pcDNA3.4 (Thermo Fisher, Waltham, MA) expression vector. Proteins were expressed by transient transfection in Expi293 cells (Thermo Fisher, Waltham, MA) according to the manufacturer’s instructions. Gp140 proteins were purified from clarified cell culture supernatants 4 days post-transfection using Galanthus nivalis lectin (GNL) affinity chromatography. When needed, gp140 proteins were further purified by flow-through on a Q Sepharose column (GE Healthcare, Chicago, IL) as a polishing step to help remove host cell protein contaminants. V1V2-gp70 scaffolds were purified from clarified cell culture supernatants 4 days post-transfection using Ni-NTA affinity resin (Qiagen, Germantown, MD). All proteins were purified to 90% purity or higher, as assessed by SDS-PAGE and Coomassie staining in reducing and non-reducing conditions.

Antibody binding and characterization were then performed as previously described (101, 104, 105) with minor modifications. Samples were assayed against a panel of four SHIV antigens (SHIV.C.CH505-gp140, SHIV.C.CH505-V1V2-gp70, SHIV-1157ipd3N4-gp140, and SHIV-1157ipd3N4-V1V2-gp70; MHRP Protein Production Lab). Antigens were coupled to uniquely coded carboxylated magnetic microspheres (Luminex Corp., Austin, TX) according to the manufacturer’s protocol and as previously described. Pooled antigens were incubated with heat-inactivated plasma for 2 h at room temperature, washed, and then incubated for 1 h with phycoerythrin-labeled non-human primate cross-reactive anti-human IgG (Southern Biotech, Birmingham, AL). After a final wash to remove unbound detection reagent, microspheres were resuspended in 40 μL sheath fluid (Luminex Corp), and data were collected on a Bio-Plex 3D Suspension Array System (Bio-Rad, Hercules, CA) running xPONENT v.4.2 (Luminex Corp). The signal from a pooled sample comprised of plasma from 10 SHIV/HIV naïve animals served as the negative control. Study samples were analyzed as fold over the negative control, and where applicable, the signal from the negative control MuLV-gp70 was subtracted to account for anti-scaffold-directed signals.

### Neutralizing antibody responses

Vaccinated animal sera were assessed for HIV-1 neutralizing activity in TZM-bl target cells as described previously (106). Pseudoviruses evaluated included C06980.v0.c22 (Tier 1, subtype C), CH505.375H, 1157ipd3N4, and an MuLV negative control.

### Antibody-dependent phagocytotic effector antibody responses

ADCP was measured as previously described (107), using CO6980v0c22 gp145, CH505.375H gp140 and V1V2, and 1157ipd3N4 gp140 and V1V2. Antigens were biotinylated following the manufacturer’s instructions (Thermo Fisher Scientific) and incubated with yellow-green streptavidin-fluorescent beads (Molecular Probes) overnight at 4°C. A 100-fold dilution of antigen-coated beads was incubated for 2 h at 37°C with diluted serum or plasma samples and washed before addition of THP-1 cells (Sigma Millipore, Cat. #88081201; 20,000 cells per well in duplicate). Following 19 h of incubation at 37°C, the cells were fixed with a 2% formaldehyde solution (Tousimis), and fluorescence was evaluated on an LSR II cytometer (BD Biosciences; representative gating tree is shown in Fig. S13a). The phagocytic score was calculated by multiplying the percentage of bead-positive cells by the geometric mean fluorescence intensity (MFI) of the bead-positive cells and dividing by 10^4^. Background responses assessed in the absence of serum or plasma were subtracted from all values.

For ADNP, CO6980v0c22 gp145, CH505.375H gp140, and 1157ipd3N4 gp140 were biotinylated following the manufacturer’s instructions (Thermo Fisher) and incubated with yellow-green streptavidin-fluorescent beads (Molecular Probes) for 2 h at 37°C. Ten microliters of a 100-fold dilution of the bead–protein conjugate was incubated for 2 h at 37°C with 100 μL of 250-fold diluted serum and washed before the addition of effector cells (50,000 cells/well). Fresh peripheral blood leukocytes were used as effector cells and prepared from human blood by red blood cell lysis with ACK lysing buffer (Thermo Fisher). After 1 h of incubation at 37°C, cells were washed, surface stained, fixed with 2% formaldehyde solution, and evaluated using an LSR II cytometer (BD Biosciences). Antibodies used for flow cytometry were as follows: CD3-Alexa Fluor 700 (UCHT1, BD Biosciences, Cat. #557943, Lot 9050801), CD14-APC-Cy7 (MϕP9, BD Biosciences, Cat. #557831, Lot 8184721), and CD66b-Pacific Blue (G10F5, BioLegend, Cat. #305112, Lot B256448). The phagocytic score was calculated by multiplying the percentage of bead-positive neutrophils (SSC high, CD3^−^ CD14^−^ CD66^+^; Fig. S13b) by the geometric MFI of the bead-positive cells and dividing by 10^4^.

### Plasma cytokine concentration

Cytokine levels were measured in plasma using the MILLIPLEX MAP Non-Human Primate Cytokine Magnetic Bead Panel–Premixed 23 Plex Luminex kit (Millipore Sigma) according to the manufacturer’s instructions. The samples were read using a Bio-Plex Magpix Multiplex Reader (Bio-Rad) machine. An asymmetric sigmoidal 5PL (X = log[concentration]) model curve with least squares fit was used to interpolate cytokine concentrations using GraphPad Prism software. Values above the limit of quantitation were assigned the upper limit of detection value, while values below the lower limit of detection were assigned a value half of this limit when determining fold change between samples.

### Envelope sequence analysis

A phylogenetic tree of the vaccine and challenge envelope sequences, along with representative envelope sequences from different clades, was built using a maximum likelihood method with an HIVb amino acid substitution matrix and optimized on a gamma distribution (108). Pairwise distances were calculated under an HIVb model in phangorn (109).

### Antigen-specific T cell responses

Cryopreserved PBMC were thawed, rested overnight in R10 with 50 U/mL Benzonase Nuclease (Sigma-Aldrich Millipore, Cat. #71205_3), and stimulated with peptide pools for 6 h. Peptides included: (i) HIV-1 Env potential T cell epitope (PTE) pools (1 µg/mL, NIH AIDS Reagent Program, Cat. #12698; either two pools of 480 peptides, used for time points at baseline and following the third immunization, or one pool of 467 peptides used for time points prior to the third immunization due to product availability); (ii) SIVmac239 Gag peptide pool (2 µg/mL, NIH AIDS Reagent Program, Cat. #12364); and (iii) HIV-1 PTE Gag peptide pool (1 µg/mL, NIH AIDS Reagent Program, Cat. #12437). Stimulations were performed in the presence of Brefeldin A (0.7 µL/mL, GolgiPlug, BD Cytofix/Cytoperm Kit, Cat. #555028). Live MVA (p581 virus in 7.5% Lactose in PBS, BPR #045.4160.1497, Lot #L132-103114-02) stimulation contained 1 PFU/cell for a total of 20 h, with Brefeldin A (0.7 µL/mL, GolgiPlug, BD, Cat. #555029) and monensin (0.35 µL/mL, GolgiStop, BD, Cat. #554724) added for the last 14 h.

Following stimulation, cells were stained serially with LIVE/DEAD Fixable Aqua (Thermo Fisher, Cat. #L34957) and a cocktail of titrated fluorescently labeled antibodies to cell surface markers. Flow cytometry panel applied to peak immunogenicity and post-infection time points contained the following anti-human antibodies: CD95-BV421 (Cat. #305624, clone DX-2), CD4 BV605 (Cat. #317438, clone OKT4), CD8 BV711 (Cat. #301044, clone RPA-T8), CCR7 BV785 (Cat. #353230, clone GO43H7), and ICOS AF700 (Cat. #313528, clone C398.4A), all from BioLegend; CD28 PE-Cy5 (Cat. #555730, clone 28.2), CD45RA BUV395 (Cat. #740315, clone 5H9), and PD-1 BUV737 (Cat. #612791, clone EH12.1), all from BD Biosciences. Intracellular cytokine staining was performed following fixation and permeabilization (BD Cytofix/Cytoperm, Becton Dickinson) with TNF FITC (Cat. #552889, clone Mab11), IFNγ APC (Cat. #551385, clone 4S.B3), CD3 APC-Cy7 (Cat. #557757, clone SP34.2), and IL-21 PE (Cat. #560463, clone 3A3-N2.1), all from BD Biosciences; IL-2 BV650 (Cat. #500334, clone MQ1-17H12) and CD154 PE-CY7 (Cat. #310832, clone 24-31), all from BioLegend; and CD69 ECD (Cat. #6607110, clone TP1.55.3) from Beckman Coulter. In secondary analyses of time points prior to the third immunization and for MVA immunity, the above panel was modified slightly with the following alternate antibodies: IL-21 Alexa Fluor 647 (Cat. #560493, clone 3A3-N2.1), CD154 BUV508 (Cat. #752857, clone 24-31), and IFNγ Alexa Fluor 700 (Cat. #506516, clone B27, BioLegend). Staining was measured on a BD FACSymphony A5 Research Cell Analyzer (BD Biosciences), and data were analyzed using FlowJo v.9.9.6 software (Tree Star, Inc.). Cytokine-positive CD4^+^ and CD8^+^ T cells were measured within pre-gated memory subsets, defined as single-positive or double-negative for CD45RA and CD28 (Fig. S14). Boolean combinations of cells expressing one or more cytokines were used to calculate the sum of antigen-specific cells. Statistical analysis and display of multicomponent distributions were performed with SPICE v6.1 (NIAID, NIH) (110). Response functionality and polyfunctionality scores were determined using COMPASS (14).

Rectal mucosa lymphocytes were isolated from rectal biopsies. Rectal tissues (biopsy specimens, 10 to 12 pinches) were treated for 30 min with HBSS-EDTA medium containing 1 mM of EDTA (Life Technologies) in calcium/magnesium-free Hank’s balanced salt solution (Gibco BRL). Following the removal of epithelium and intraepithelial lymphocytes, tissues were digested by shaking at 37°C with RPMI 1640 supplemented with 5% fetal bovine serum (FBS), 2 mM L-glutamine, and penicillin-streptomycin (RPMI5), plus collagenase IV (1 mg/mL; Sigma-Aldrich) for 1 h, changing to fresh RPMI5 plus collagenase after 30 min. Digested tissue was pressed twice through a 100 μm pore size filter and a 70 μm pore size filter. HIV-specific CD4^+^ and CD8^+^ T cell responses were detected using HIV-1 PTE Env Pool 1 and Pool 2 (NIH AIDS Research and Reference Reagent Program). Cells (1 × 10^6^) in RPMI 1640 medium (containing 10% fetal bovine serum and antibiotics) were incubated with and without peptide pools for 1 h or in the presence of the phorbol 12-myristate 13-acetate (PMA; 10 ng/mL) plus Ionomycin (1 μg/mL; Sigma) as a positive control. Peptide diluent (1% DMSO) served as the negative control. Co-stimulatory antibodies to CD28 and CD49d (0.5 μg/mL; BD Pharmingen) were added to all the samples to maximize the detection of T cells with higher activation thresholds. GolgiPlug (1 μg/mL; BD Biosciences) was added to all cultures, which were then incubated for an additional 10–12 h. The cells were washed and stained for 15 min using LIVE/DEAD-Pacific Blue (Life Technologies), followed by surface staining with the following anti-human antibodies: CD3-Alexa Fluor 700 (BD Biosciences), CD8 APC-C7 (BD Biosciences), CD4 BV605 (BD Biosciences), CD154 APC (BioLegend), and CD195 PerCP-Cy5.5 (CCR5) (Beckman Coulter). Cells were permeabilized with Caltag Fixation and Permeabilization Kit (Life Technologies) and stained intracellularly with IL-2 PE (BD Biosciences), IFNγ PE Cy7 (BD Biosciences), CD69 Texas Red (BD Biosciences), and TNFα-FITC (BD Biosciences). Fluorescence was measured by flow cytometry on a Fortessa (BD Biosciences) or the MACSQuant Analyzer 16 (Miltenyi Biotec) (Fig. S15).

### Binding of antibodies and plasma to cell surface-expressed Env

293T human embryonic kidney cells (ATCC) and CD4^+^ T lymphocytes were isolated, activated, and cultured as previously described (111). CD4^+^ lymphocytes were isolated from rhesus macaque PBMCs (kindly provided by Matthew Parsons, MHRP) using EasySep Non-Human Primate CD4^+^ T Cell Isolation Kit (StemCell Technologies, Cat. #19582) and activated with T Cell Activation/Expansion Kit for non-human primate (Miltenyi Biotec, Cat. #130-092-919) for 48 h. Cells were maintained in complete RPMI 1640 medium supplemented with rIL-2 (100 U/mL) until infection.

For *in vitro* infection, vesicular stomatitis virus G (VSV-G)-pseudotyped SHIV viruses were produced by co-transfection of 293T cells with a SHIV proviral construct and a VSV-G-encoding vector using polycation polyethylenimine (PEI). We used VSV-G pseudotypes to ensure a high frequency of infected (p27^+^) cells for analysis, as VSV-G improves entry but does not affect the expression, localization, or trafficking of the newly synthesized Env. Two days post-transfection, cell supernatants were harvested, clarified by low-speed centrifugation (300 × *g* for 5 min), and concentrated by ultracentrifugation at 4°C (100,605 × *g* for 1 h) over a 20% sucrose cushion. Pellets were resuspended in fresh RPMI, and aliquots were stored at −80°C until use. Viruses were then used to infect activated NHP primary CD4^+^ T cells by spin infection at 800 × *g* for 1 h in 96-well plates at 25°C. All experiments using VSV-G-pseudotyped SHIV isolates were conducted in a biosafety level 3 laboratory following manipulation protocols approved by the CRCHUM Biosafety Committee, which complies with the requirements of the Public Health Agency of Canada.

Cell surface staining was performed at 48 h post-transfection of SHIV proviruses in 293T cells or post-infection in macaque primary CD4^+^ T cells with SHIV-1157ipd3N4. Mock, transfected, or infected cells were incubated for 45 min at 37°C with anti-Env mAbs (5 µg/mL) or plasma from people living with HIV-1 (PLWH) (1:1,000 dilution, obtained from the FRQS-AIDS and Infectious Diseases Network, the Montreal Primary HIV-1 Infection Cohort) (112, 113)). Cells were then washed twice with PBS and stained with the appropriate Alexa Fluor 647-conjugated secondary antibody (2 µg/mL, Thermo Fisher Scientific) and the viability dye AquaVivid viability (Thermo Fisher Scientific) for 20 min at room temperature, followed with the staining with PE-conjugated mouse anti-human CD4 (Clone OKT4) antibody (1:500 dilution) for 20 min. Cells were then washed twice with PBS and fixed in a 2% PBS-formaldehyde solution. Infected cells were permeabilized using the Cytofix/Cytoperm Fixation/Permeabilization Kit (BD Biosciences) and stained intracellularly using FITC-conjugated mouse anti-p24 mAb (clone KC57; Beckman Coulter; 1:100 dilution). Samples were acquired on an LSR II cytometer (BD Biosciences), and data analysis was performed using FlowJo v10.5.3 (Tree Star).

### Statistical analysis

Survival analysis was performed to assess the effect of different vaccine groups on the time to infection. The discrete infecting challenge (i.e., 1, 2, …, 10) was treated as the time-to-event data. A Cox proportional hazards model was utilized to compare the hazard rates among the different vaccine groups. This semi-parametric model allows estimation of hazard ratios without making assumptions about the shape or scale of the underlying survival distribution. The model was fit using R’s survival package in R environment (version 4.3.1) with “Infecting challenge” as the response variable and “Vaccine group” as the predictor. The reference group for these analyses was set as Sham.

Statistical comparisons of immunological and virological outcomes between groups were performed using GraphPad Prism v9.4.1 software. Primary immunogenicity outputs were compared across vaccination groups using a non-parametric Mann-Whitney test. A non-parametric permutation test was performed to compare the CD4^+^ T cell response functionality between vaccine arms in SPICE. Post-vaccination comparisons were made across all three groups using a Kruskal-Wallis test. Non-parametric pairwise comparisons between groups were made using the Dunn’s post-hoc test. Statistical significance was set at an alpha level of 0.05.

## Data Availability

The data sets used and/or analyzed during the current study are available from the corresponding author on reasonable request.

## SUPPLEMENTAL MATERIAL

Supplemental material jvi.00389-26-s0001.pdf Fig. S1 to S15.

ASM does not own the copyrights to Supplemental Material that may be linked to, or accessed through, an article. The authors have granted ASM a non-exclusive, world-wide license to publish the Supplemental Material files. Please contact the corresponding author directly for reuse.

## References

[1] UNAIDS. 2024. Global HIV & AIDS statistics — fact sheet. Available from: https://www.unaids.org/en/resources/fact-sheet

[2] Pitisuttithum P, Gilbert P, Gurwith M, Heyward W, Martin M, van Griensven F, Hu D, Tappero JW, Choopanya K. 2006. Randomized, double-blind, placebo-controlled efficacy trial of a bivalent recombinant glycoprotein 120 HIV-1 vaccine among injection drug users in Bangkok, Thailand. J Infect Dis 194:1661–1671. doi:10.1086/508748 17109337

[3] Flynn NM, Forthal DN, Harro CD, Judson FN, Mayer KH, Para MF. 2005. Placebo-controlled phase 3 trial of a recombinant glycoprotein 120 vaccine to prevent HIV-1 infection. J Infect Dis 191:654–665. doi:10.1086/428404 15688278

[4] Buchbinder SP, Mehrotra DV, Duerr A, Fitzgerald DW, Mogg R, Li D, Gilbert PB, Lama JR, Marmor M, del Rio C, McElrath MJ, Casimiro DR, Gottesdiener KM, Chodakewitz JA, Corey L, Robertson MN. 2008. Efficacy assessment of a cell-mediated immunity HIV-1 vaccine (the Step Study): a double-blind, randomised, placebo-controlled, test-of-concept trial. The Lancet 372:1881–1893. doi:10.1016/S0140-6736(08)61591-3 PMC2721012 19012954

[5] Gray GE, Allen M, Moodie Z, Churchyard G, Bekker L-G, Nchabeleng M, Mlisana K, Metch B, de Bruyn G, Latka MH, Roux S, Mathebula M, Naicker N, Ducar C, Carter DK, Puren A, Eaton N, McElrath MJ, Robertson M, Corey L, Kublin JG. 2011. Safety and efficacy of the HVTN 503/Phambili Study of a clade-B-based HIV-1 vaccine in South Africa: a double-blind, randomised, placebo-controlled test-of-concept phase 2b study. Lancet Infect Dis 11:507–515. doi:10.1016/S1473-3099(11)70098-6 21570355 PMC3417349

[6] Hammer SM, Sobieszczyk ME, Janes H, Karuna ST, Mulligan MJ, Grove D, Koblin BA, Buchbinder SP, Keefer MC, Tomaras GD, et al.. 2013. Efficacy trial of a DNA/rAd5 HIV-1 preventive vaccine. N Engl J Med 369:2083–2092. doi:10.1056/NEJMoa1310566 24099601 PMC4030634

[7] The Lancet HIV. 2023. What future for HIV vaccines? Lancet HIV 10:e143. doi:10.1016/S2352-3018(23)00030-9 36803798

[8] Rerks-Ngarm S, Pitisuttithum P, Nitayaphan S, Kaewkungwal J, Chiu J, Paris R, Premsri N, Namwat C, de Souza M, Adams E, Benenson M, Gurunathan S, Tartaglia J, McNeil JG, Francis DP, Stablein D, Birx DL, Chunsuttiwat S, Khamboonruang C, Thongcharoen P, Robb ML, Michael NL, Kunasol P, Kim JH. 2009. Vaccination with ALVAC and AIDSVAX to prevent HIV-1 infection in Thailand. N Engl J Med 361:2209–2220. doi:10.1056/NEJMoa0908492 19843557

[9] Robb ML, Rerks-Ngarm S, Nitayaphan S, Pitisuttithum P, Kaewkungwal J, Kunasol P, Khamboonruang C, Thongcharoen P, Morgan P, Benenson M, Paris RM, Chiu J, Adams E, Francis D, Gurunathan S, Tartaglia J, Gilbert P, Stablein D, Michael NL, Kim JH. 2012. Risk behaviour and time as covariates for efficacy of the HIV vaccine regimen ALVAC-HIV (vCP1521) and AIDSVAX B/E: a post-hoc analysis of the Thai phase 3 efficacy trial RV 144. Lancet Infect Dis 12:531–537. doi:10.1016/S1473-3099(12)70088-9 22652344 PMC3530398

[10] Gray GE, Bekker L-G, Laher F, Malahleha M, Allen M, Moodie Z, Grunenberg N, Huang Y, Grove D, Prigmore B, et al.. 2021. Vaccine efficacy of ALVAC-HIV and bivalent subtype C gp120-MF59 in adults. N Engl J Med 384:1089–1100. doi:10.1056/NEJMoa2031499 33761206 PMC7888373

[11] Zolla-Pazner S, Michael NL, Kim JH. 2021. A tale of four studies: HIV vaccine immunogenicity and efficacy in clinical trials. Lancet HIV 8:e449–e452. doi:10.1016/S2352-3018(21)00073-4 34029515 PMC8449712

[12] Haynes BF, Gilbert PB, McElrath MJ, Zolla-Pazner S, Tomaras GD, Alam SM, Evans DT, Montefiori DC, Karnasuta C, Sutthent R, et al.. 2012. Immune-correlates analysis of an HIV-1 vaccine efficacy trial. N Engl J Med 366:1275–1286. doi:10.1056/NEJMoa1113425 22475592 PMC3371689

[13] Chung AW, Ghebremichael M, Robinson H, Brown E, Choi I, Lane S, Dugast A-S, Schoen MK, Rolland M, Suscovich TJ, Mahan AE, Liao L, Streeck H, Andrews C, Rerks-Ngarm S, Nitayaphan S, de Souza MS, Kaewkungwal J, Pitisuttithum P, Francis D, Michael NL, Kim JH, Bailey-Kellogg C, Ackerman ME, Alter G. 2014. Polyfunctional Fc-effector profiles mediated by IgG subclass selection distinguish RV144 and VAX003 vaccines. Sci Transl Med 6:228ra38. doi:10.1126/scitranslmed.3007736 24648341

[14] Lin L, Finak G, Ushey K, Seshadri C, Hawn TR, Frahm N, Scriba TJ, Mahomed H, Hanekom W, Bart P-A, Pantaleo G, Tomaras GD, Rerks-Ngarm S, Kaewkungwal J, Nitayaphan S, Pitisuttithum P, Michael NL, Kim JH, Robb ML, O’Connell RJ, Karasavvas N, Gilbert P, C De Rosa S, McElrath MJ, Gottardo R. 2015. COMPASS identifies T-cell subsets correlated with clinical outcomes. Nat Biotechnol 33:610–616. doi:10.1038/nbt.3187 26006008 PMC4569006

[15] Neidich SD, Fong Y, Li SS, Geraghty DE, Williamson BD, Young WC, Goodman D, Seaton KE, Shen X, Sawant S, et al.. 2019. Antibody Fc effector functions and IgG3 associate with decreased HIV-1 risk. J Clin Invest 129:4838–4849. doi:10.1172/JCI126391 31589165 PMC6819135

[16] Roederer M, Keele BF, Schmidt SD, Mason RD, Welles HC, Fischer W, Labranche C, Foulds KE, Louder MK, Yang Z-Y, et al.. 2014. Immunological and virological mechanisms of vaccine-mediated protection against SIV and HIV. Nature 505:502–508. doi:10.1038/nature12893 24352234 PMC3946913

[17] Barouch DH, Stephenson KE, Borducchi EN, Smith K, Stanley K, McNally AG, Liu J, Abbink P, Maxfield LF, Seaman MS, et al.. 2013. Protective efficacy of a global HIV-1 mosaic vaccine against heterologous SHIV challenges in rhesus monkeys. Cell 155:531–539. doi:10.1016/j.cell.2013.09.061 24243013 PMC3846288

[18] Bogers WMJM, Davis D, Baak I, Kan E, Hofman S, Sun Y, Mortier D, Lian Y, Oostermeijer H, Fagrouch Z, Dubbes R, van der Maas M, Mooij P, Koopman G, Verschoor E, Langedijk JPM, Zhao J, Brocca-Cofano E, Robert-Guroff M, Srivastava I, Barnett S, Heeney JL. 2008. Systemic neutralizing antibodies induced by long interval mucosally primed systemically boosted immunization correlate with protection from mucosal SHIV challenge. Virology 382:217–225. doi:10.1016/j.virol.2008.09.016 18947849 PMC2723753

[19] Arunachalam PS, Charles TP, Joag V, Bollimpelli VS, Scott MKD, Wimmers F, Burton SL, Labranche CC, Petitdemange C, Gangadhara S, et al.. 2020. T cell-inducing vaccine durably prevents mucosal SHIV infection even with lower neutralizing antibody titers. Nat Med 26:932–940. doi:10.1038/s41591-020-0858-8 32393800 PMC7303014

[20] Pauthner MG, Nkolola JP, Havenar-Daughton C, Murrell B, Reiss SM, Bastidas R, Prévost J, Nedellec R, von Bredow B, Abbink P, et al.. 2019. Vaccine-induced protection from homologous Tier 2 SHIV challenge in nonhuman primates depends on serum-neutralizing antibody titers. Immunity 50:241–252. doi:10.1016/j.immuni.2018.11.011 30552025 PMC6335502

[21] Zhang P, Narayanan E, Liu Q, Tsybovsky Y, Boswell K, Ding S, Hu Z, Follmann D, Lin Y, Miao H, et al.. 2021. A multiclade env–gag VLP mRNA vaccine elicits tier-2 HIV-1-neutralizing antibodies and reduces the risk of heterologous SHIV infection in macaques. Nat Med 27:2234–2245. doi:10.1038/s41591-021-01574-5 34887575

[22] Corey L, Gilbert PB, Juraska M, Montefiori DC, Morris L, Karuna ST, Edupuganti S, Mgodi NM, deCamp AC, Rudnicki E, et al.. 2021. Two randomized trials of neutralizing antibodies to prevent HIV-1 acquisition. N Engl J Med 384:1003–1014. doi:10.1056/NEJMoa2031738 33730454 PMC8189692

[23] Felber BK, Lu Z, Hu X, Valentin A, Rosati M, Remmel CAL, Weiner JA, Carpenter MC, Faircloth K, Stanfield-Oakley S, et al.. 2020. Co-immunization of DNA and protein in the same anatomical sites induces superior protective immune responses against SHIV challenge. Cell Rep 31:107624. doi:10.1016/j.celrep.2020.107624 32402293 PMC7329227

[24] Barouch DH, Liu J, Li H, Maxfield LF, Abbink P, Lynch DM, Iampietro MJ, SanMiguel A, Seaman MS, Ferrari G, et al.. 2012. Vaccine protection against acquisition of neutralization-resistant SIV challenges in rhesus monkeys. Nature 482:89–93. doi:10.1038/nature10766 22217938 PMC3271177

[25] Barouch DH, Tomaka FL, Wegmann F, Stieh DJ, Alter G, Robb ML, Michael NL, Peter L, Nkolola JP, Borducchi EN, et al.. 2018. Evaluation of a mosaic HIV-1 vaccine in a multicentre, randomised, double-blind, placebo-controlled, phase 1/2a clinical trial (APPROACH) and in rhesus monkeys (NHP 13-19). Lancet 392:232–243. doi:10.1016/S0140-6736(18)31364-3 30047376 PMC6192527

[26] Barouch DH, Alter G, Broge T, Linde C, Ackerman ME, Brown EP, Borducchi EN, Smith KM, Nkolola JP, Liu J, et al.. 2015. Protective efficacy of adenovirus/protein vaccines against SIV challenges in rhesus monkeys. Science 349:320–324. doi:10.1126/science.aab3886 26138104 PMC4653134

[27] Ackerman ME, Das J, Pittala S, Broge T, Linde C, Suscovich TJ, Brown EP, Bradley T, Natarajan H, Lin S, Sassic JK, O’Keefe S, Mehta N, Goodman D, Sips M, Weiner JA, Tomaras GD, Haynes BF, Lauffenburger DA, Bailey-Kellogg C, Roederer M, Alter G. 2018. Route of immunization defines multiple mechanisms of vaccine-mediated protection against SIV. Nat Med 24:1590–1598. doi:10.1038/s41591-018-0161-0 30177821 PMC6482471

[28] Om K, Paquin-Proulx D, Montero M, Peachman K, Shen X, Wieczorek L, Beck Z, Weiner JA, Kim D, Li Y, et al.. 2020. Adjuvanted HIV-1 vaccine promotes antibody-dependent phagocytic responses and protects against heterologous SHIV challenge. PLoS Pathog 16:e1008764. doi:10.1371/journal.ppat.1008764 32881968 PMC7505435

[29] Bradley T, Pollara J, Santra S, Vandergrift N, Pittala S, Bailey-Kellogg C, Shen X, Parks R, Goodman D, Eaton A, et al.. 2017. Pentavalent HIV-1 vaccine protects against simian-human immunodeficiency virus challenge. Nat Commun 8:15711. doi:10.1038/ncomms15711 28593989 PMC5472724

[30] Fouts TR, Bagley K, Prado IJ, Bobb KL, Schwartz JA, Xu R, Zagursky RJ, Egan MA, Eldridge JH, LaBranche CC, Montefiori DC, Le Buanec H, Zagury D, Pal R, Pavlakis GN, Felber BK, Franchini G, Gordon S, Vaccari M, Lewis GK, DeVico AL, Gallo RC. 2015. Balance of cellular and humoral immunity determines the level of protection by HIV vaccines in rhesus macaque models of HIV infection. Proc Natl Acad Sci USA 112. doi:10.1073/pnas.1423669112 PMC4352796 25681373

[31] Bissa M, Kim S, Galli V, Fourati S, Sarkis S, Arakelyan A, de Castro IS, Rahman MA, Fujiwara S, Vaccari M, et al.. 2023. HIV vaccine candidate efficacy in female macaques mediated by cAMP-dependent efferocytosis and V2-specific ADCC. Nat Commun 14:575. doi:10.1038/s41467-023-36109-8 36732510 PMC9894672

[32] Di Pasquale A, Preiss S, Tavares Da Silva F, Garçon N. 2015. Vaccine adjuvants: from 1920 to 2015 and beyond. Vaccines (Basel) 3:320–343. doi:10.3390/vaccines3020320 26343190 PMC4494348

[33] Didierlaurent AM, Morel S, Lockman L, Giannini SL, Bisteau M, Carlsen H, Kielland A, Vosters O, Vanderheyde N, Schiavetti F, Larocque D, Van Mechelen M, Garçon N. 2009. AS04, an aluminum salt- and TLR4 agonist-based adjuvant system, induces a transient localized innate immune response leading to enhanced adaptive immunity. J Immunol 183:6186–6197. doi:10.4049/jimmunol.0901474 19864596

[34] Alving CR, Peachman KK, Matyas GR, Rao M, Beck Z. 2020. Army liposome formulation (ALF) family of vaccine adjuvants. Expert Rev Vaccines 19:279–292. doi:10.1080/14760584.2020.1745636 32228108 PMC7412170

[35] King HAD, Joyce MG, Lakhal-Naouar I, Ahmed A, Cincotta CM, Subra C, Peachman KK, Hack HR, Chen RE, Thomas PV, et al.. 2021. Efficacy and breadth of adjuvanted SARS-CoV-2 receptor-binding domain nanoparticle vaccine in macaques. Proc Natl Acad Sci USA 118. doi:10.1073/pnas.2106433118 PMC8463842 34470866

[36] Johnston SC, Ricks KM, Lakhal-Naouar I, Jay A, Subra C, Raymond JL, King HAD, Rossi F, Clements TL, Fetterer D, et al.. 2022. A SARS-CoV-2 spike ferritin nanoparticle vaccine is protective and promotes a strong immunological response in the cynomolgus macaque coronavirus disease 2019 (COVID-19) model. Vaccines (Basel) 10:717. doi:10.3390/vaccines10050717 35632473 PMC9145473

[37] Joyce MG, Chen W-H, Sankhala RS, Hajduczki A, Thomas PV, Choe M, Martinez EJ, Chang WC, Peterson CE, Morrison EB, et al.. 2021. SARS-CoV-2 ferritin nanoparticle vaccines elicit broad SARS coronavirus immunogenicity. Cell Rep 37:110143. doi:10.1016/j.celrep.2021.110143 34919799 PMC8651551

[38] Ober Shepherd BL, Scott PT, Hutter JN, Lee C, McCauley MD, Guzman I, Bryant C, McGuire S, Kennedy J, Chen W-H, et al.. 2024. SARS-CoV-2 recombinant spike ferritin nanoparticle vaccine adjuvanted with army liposome formulation containing monophosphoryl lipid A and QS-21: a phase 1, randomised, double-blind, placebo-controlled, first-in-human clinical trial. The Lancet Microbe 5:e581–e593. doi:10.1016/S2666-5247(23)00410-X 38761816 PMC11192176

[39] Joyce MG, King HAD, Elakhal-Naouar I, Ahmed A, Peachman KK, Macedo Cincotta C, Subra C, Chen RE, Thomas PV, Chen W-H, et al.. 2022. A SARS-CoV-2 ferritin nanoparticle vaccine elicits protective immune responses in nonhuman primates. Sci Transl Med 14. doi:10.1126/scitranslmed.abi5735 34914540

[40] Ramakrishnan A, Schumack NM, Gariepy CL, Eggleston H, Nunez G, Espinoza N, Nieto M, Castillo R, Rojas J, McCoy AJ, Beck Z, Matyas GR, Alving CR, Guerry P, Poly F, Laird RM. 2019. Enhanced immunogenicity and protective efficacy of a *Campylobacter jejuni* conjugate vaccine coadministered with liposomes containing monophosphoryl lipid A and QS-21. mSphere 4:e00101-19. doi:10.1128/mSphere.00101-19 31043512 PMC6495334

[41] Rikhi N, Sei CJ, Rao M, Schuman RF, Kroscher KA, Matyas GR, Muema K, Lange C, Assiaw-Dufu A, Hussin E, Jobe O, Alving CR, Fischer GW. 2023. Unconjugated multi-epitope peptides adjuvanted with ALFQ induce durable and broadly reactive antibodies to human and avian influenza viruses. Vaccines (Basel) 11:1468. doi:10.3390/vaccines11091468 37766144 PMC10537791

[42] Scaria PV, Anderson C, Muratova O, Alani N, Trinh HV, Nadakal ST, Zaidi I, Lambert L, Beck Z, Barnafo EK, Rausch KM, Rowe C, Chen B, Matyas GR, Rao M, Alving CR, Narum DL, Duffy PE. 2021. Malaria transmission-blocking conjugate vaccine in ALFQ adjuvant induces durable functional immune responses in rhesus macaques. NPJ Vaccines 6:148. doi:10.1038/s41541-021-00407-3 34887448 PMC8660773

[43] Verma A, Schmidt BA, Elizaldi SR, Nguyen NK, Walter KA, Beck Z, Trinh HV, Dinasarapu AR, Lakshmanappa YS, Rane NN, Matyas GR, Rao M, Shen X, Tomaras GD, LaBranche CC, Reimann KA, Foehl DH, Gach JS, Forthal DN, Kozlowski PA, Amara RR, Iyer SS. 2020. Impact of T _h_ 1 CD4 follicular helper T cell skewing on antibody responses to an HIV-1 vaccine in rhesus macaques. J Virol 94. doi:10.1128/JVI.01737-19 PMC7158739 31827000

[44] Hutter JN, Robben PM, Lee C, Hamer M, Moon JE, Merino K, Zhu L, Galli H, Quinn X, Brown DR, Duncan E, Bolton J, Zou X, Angov E, Lanar DE, Rao M, Matyas GR, Beck Z, Bergmann-Leitner E, Soisson LA, Waters NC, Ngauy V, Regules J, Dutta S. 2022. First-in-human assessment of safety and immunogenicity of low and high doses of *Plasmodium falciparum* malaria protein 013 (FMP013) administered intramuscularly with ALFQ adjuvant in healthy malaria-naïve adults. Vaccine 40:5781–5790. doi:10.1016/j.vaccine.2022.08.048 36055874

[45] Earl PL, Cotter C, Moss B, VanCott T, Currier J, Eller LA, McCutchan F, Birx DL, Michael NL, Marovich MA, Robb M, Cox JH. 2009. Design and evaluation of multi-gene, multi-clade HIV-1 MVA vaccines. Vaccine 27:5885–5895. doi:10.1016/j.vaccine.2009.07.039 19654066 PMC2743792

[46] Wieczorek L, Krebs SJ, Kalyanaraman V, Whitney S, Tovanabutra S, Moscoso CG, Sanders-Buell E, Williams C, Slike B, Molnar S, et al.. 2015. Comparable antigenicity and immunogenicity of oligomeric forms of a novel, acute HIV-1 subtype C gp145 envelope for use in preclinical and clinical vaccine research. J Virol 89:7478–7493. doi:10.1128/JVI.00412-15 25972551 PMC4505676

[47] Bar KJ, Coronado E, Hensley-McBain T, O’Connor MA, Osborn JM, Miller C, Gott TM, Wangari S, Iwayama N, Ahrens CY, Smedley J, Moats C, Lynch RM, Haddad EK, Haigwood NL, Fuller DH, Shaw GM, Klatt NR, Manuzak JA. 2019. Simian-human immunodeficiency virus SHIV.CH505 infection of rhesus macaques results in persistent viral replication and induces intestinal immunopathology. J Virol 93:00372–19. doi:10.1128/JVI.00372-19 PMC6714786 31217249

[48] Li H, Wang S, Kong R, Ding W, Lee F-H, Parker Z, Kim E, Learn GH, Hahn P, Policicchio B, et al.. 2016. Envelope residue 375 substitutions in simian–human immunodeficiency viruses enhance CD4 binding and replication in rhesus macaques. Proc Natl Acad Sci USA 113. doi:10.1073/pnas.1606636113 PMC4914158 27247400

[49] Song RJ, Chenine AL, Rasmussen RA, Ruprecht CR, Mirshahidi S, Grisson RD, Xu W, Whitney JB, Goins LM, Ong H, Li PL, Shai-Kobiler E, Wang T, McCann CM, Zhang H, Wood C, Kankasa C, Secor WE, McClure HM, Strobert E, Else JG, Ruprecht RM. 2006. Molecularly cloned SHIV-1157ipd3N4: a highly replication- competent, mucosally transmissible R5 simian-human immunodeficiency virus encoding HIV clade C Env. J Virol 80:8729–8738. doi:10.1128/JVI.00558-06 16912320 PMC1563858

[50] Liao H-X, Lynch R, Zhou T, Gao F, Alam SM, Boyd SD, Fire AZ, Roskin KM, Schramm CA, Zhang Z, et al.. 2013. Co-evolution of a broadly neutralizing HIV-1 antibody and founder virus. Nature 496:469–476. doi:10.1038/nature12053 23552890 PMC3637846

[51] Akapirat S, Karnasuta C, Vasan S, Rerks-Ngarm S, Pitisuttithum P, Madnote S, Savadsuk H, Rittiroongrad S, Puangkaew J, Phogat S, Tartaglia J, Sinangil F, de Souza MS, Excler J-L, Kim JH, Robb ML, Michael NL, Ngauy V, O’Connell RJ, Karasavvas N, Group RVS. 2018. Characterization of HIV-1 gp120 antibody specificities induced in anogenital secretions of RV144 vaccine recipients after late boost immunizations. PLoS One 13:e0196397. doi:10.1371/journal.pone.0196397 29702672 PMC5922559

[52] Li H, Stephenson KE, Kang ZH, Lavine CL, Seaman MS, Barouch DH. 2014. Common features of mucosal and peripheral antibody responses elicited by candidate HIV-1 vaccines in rhesus monkeys. J Virol 88:13510–13515. doi:10.1128/JVI.02095-14 25210178 PMC4249108

[53] Scherpenisse M, Mollers M, Schepp RM, Meijer CJLM, de Melker HE, Berbers GAM, van der Klis FRM. 2013. Detection of systemic and mucosal HPV-specific IgG and IgA antibodies in adolescent girls one and two years after HPV vaccination. Hum Vaccin Immunother 9:314–321. doi:10.4161/hv.22693 23149693 PMC3859753

[54] Spiekermann GM, Finn PW, Ward ES, Dumont J, Dickinson BL, Blumberg RS, Lencer WI. 2002. Receptor-mediated immunoglobulin G transport across mucosal barriers in adult life: functional expression of FcRn in the mammalian lung. J Exp Med 196:303–310. doi:10.1084/jem.20020400 12163559 PMC2193935

[55] Elgueta R, Benson MJ, de Vries VC, Wasiuk A, Guo Y, Noelle RJ. 2009. Molecular mechanism and function of CD40/CD40L engagement in the immune system. Immunol Rev 229:152–172. doi:10.1111/j.1600-065X.2009.00782.x 19426221 PMC3826168

[56] Corey L, Gilbert PB, Tomaras GD, Haynes BF, Pantaleo G, Fauci AS. 2015. Immune correlates of vaccine protection against HIV-1 acquisition. Sci Transl Med 7:310rv7. doi:10.1126/scitranslmed.aac7732 PMC4751141 26491081

[57] Darrah PA, Patel DT, De Luca PM, Lindsay RWB, Davey DF, Flynn BJ, Hoff ST, Andersen P, Reed SG, Morris SL, Roederer M, Seder RA. 2007. Multifunctional TH1 cells define a correlate of vaccine-mediated protection against *Leishmania* major. Nat Med 13:843–850. doi:10.1038/nm1592 17558415

[58] Saunders KO, Pardi N, Parks R, Santra S, Mu Z, Sutherland L, Scearce R, Barr M, Eaton A, Hernandez G, et al.. 2021. Lipid nanoparticle encapsulated nucleoside-modified mRNA vaccines elicit polyfunctional HIV-1 antibodies comparable to proteins in nonhuman primates. NPJ Vaccines 6:50. doi:10.1038/s41541-021-00307-6 33837212 PMC8035178

[59] Yang R, Masters AR, Fortner KA, Champagne DP, Yanguas-Casás N, Silberger DJ, Weaver CT, Haynes L, Rincon M. 2016. IL-6 promotes the differentiation of a subset of naive CD8+ T cells into IL-21-producing B helper CD8+ T cells. J Exp Med 213:2281–2291. doi:10.1084/jem.20160417 27670591 PMC5068236

[60] Gabay C, Lamacchia C, Palmer G. 2010. IL-1 pathways in inflammation and human diseases. Nat Rev Rheumatol 6:232–241. doi:10.1038/nrrheum.2010.4 20177398

[61] Ivashkiv LB. 2018. IFNγ: signalling, epigenetics and roles in immunity, metabolism, disease and cancer immunotherapy. Nat Rev Immunol 18:545–558. doi:10.1038/s41577-018-0029-z 29921905 PMC6340644

[62] Ihim SA, Abubakar SD, Zian Z, Sasaki T, Saffarioun M, Maleknia S, Azizi G. 2022. Interleukin-18 cytokine in immunity, inflammation, and autoimmunity: Biological role in induction, regulation, and treatment. Front Immunol 13. doi:10.3389/fimmu.2022.919973 PMC9410767 36032110

[63] Deshmane SL, Kremlev S, Amini S, Sawaya BE. 2009. Monocyte chemoattractant protein-1 (MCP-1): an overview. J Interferon Cytokine Res 29:313–326. doi:10.1089/jir.2008.0027 19441883 PMC2755091

[64] Cox MA, Kahan SM, Zajac AJ. 2013. Anti-viral CD8 T cells and the cytokines that they love. Virology 435:157–169. doi:10.1016/j.virol.2012.09.012 23217625 PMC3580945

[65] Chan YN, Boesch AW, Osei-Owusu NY, Emileh A, Crowley AR, Cocklin SL, Finstad SL, Linde CH, Howell RA, Zentner I, Cocklin S, Miles AR, Eckman JW, Alter G, Schmitz JE, Ackerman ME. 2016. IgG binding characteristics of rhesus macaque FcγR. J Immunol 197:2936–2947. doi:10.4049/jimmunol.1502252 27559046 PMC5026948

[66] Richard J, Prévost J, Alsahafi N, Ding S, Finzi A. 2018. Impact of HIV-1 envelope conformation on ADCC responses. Trends Microbiol 26:253–265. doi:10.1016/j.tim.2017.10.007 29162391

[67] McLellan JS, Pancera M, Carrico C, Gorman J, Julien J-P, Khayat R, Louder R, Pejchal R, Sastry M, Dai K, et al.. 2011. Structure of HIV-1 gp120 V1/V2 domain with broadly neutralizing antibody PG9. Nature 480:336–343. doi:10.1038/nature10696 22113616 PMC3406929

[68] Falkowska E, Le KM, Ramos A, Doores KJ, Lee JH, Blattner C, Ramirez A, Derking R, van Gils MJ, Liang C-H, et al.. 2014. Broadly neutralizing HIV antibodies define a glycan-dependent epitope on the prefusion conformation of gp41 on cleaved envelope trimers. Immunity 40:657–668. doi:10.1016/j.immuni.2014.04.009 24768347 PMC4070425

[69] Wang Y-Q, Bazin-Lee H, Evans JT, Casella CR, Mitchell TC. 2020. MPL adjuvant contains competitive antagonists of human TLR4. Front Immunol 11:577823. doi:10.3389/fimmu.2020.577823 33178204 PMC7596181

[70] Wang P. 2021. Natural and synthetic saponins as vaccine adjuvants. Vaccines (Basel) 9:222. doi:10.3390/vaccines9030222 33807582 PMC8001307

[71] Bolton DL, Song K, Wilson RL, Kozlowski PA, Tomaras GD, Keele BF, Lovingood RV, Rao S, Roederer M. 2012. Comparison of systemic and mucosal vaccination: impact on intravenous and rectal SIV challenge. Mucosal Immunol 5:41–52. doi:10.1038/mi.2011.45 22031182 PMC3732474

[72] van Duijn J, Stieh D, Fernandez N, King D, Gilmour J, Tolboom J, Callewaert K, Willems W, Pau MG, De Rosa SC, McElrath MJ, Barouch DH, Hayes P. 2023. Mosaic HIV-1 vaccination induces anti-viral CD8^+^ T cell functionality in the phase 1/2a clinical trial APPROACH. J Virol 97:e0112623. doi:10.1128/jvi.01126-23 37811993 PMC10617392

[73] Borthwick N, Ahmed T, Ondondo B, Hayes P, Rose A, Ebrahimsa U, Hayton EJ, Black A, Bridgeman A, Rosario M, Hill AV, Berrie E, Moyle S, Frahm N, Cox J, Colloca S, Nicosia A, Gilmour J, McMichael AJ, Dorrell L, Hanke T. 2014. Vaccine-elicited human T cells recognizing conserved protein regions inhibit HIV-1. Mol Ther 22:464–475. doi:10.1038/mt.2013.248 24166483 PMC3911893

[74] Mothe B, Hu X, Llano A, Rosati M, Olvera A, Kulkarni V, Valentin A, Alicea C, Pilkington GR, Sardesai NY, Rocafort M, Crespo M, Carrillo J, Marco A, Mullins JI, Dorrell L, Hanke T, Clotet B, Pavlakis GN, Felber BK, Brander C. 2015. A human immune data-informed vaccine concept elicits strong and broad T-cell specificities associated with HIV-1 control in mice and macaques. J Transl Med 13:60. doi:10.1186/s12967-015-0392-5 25879820 PMC4336696

[75] Duewell P, Kisser U, Heckelsmiller K, Hoves S, Stoitzner P, Koernig S, Morelli AB, Clausen BE, Dauer M, Eigler A, Anz D, Bourquin C, Maraskovsky E, Endres S, Schnurr M. 2011. ISCOMATRIX adjuvant combines immune activation with antigen delivery to dendritic cells in vivo leading to effective cross-priming of CD8+ T cells. J Immunol 187:55–63. doi:10.4049/jimmunol.1004114 21613613 PMC4285562

[76] Didierlaurent AM, Collignon C, Bourguignon P, Wouters S, Fierens K, Fochesato M, Dendouga N, Langlet C, Malissen B, Lambrecht BN, Garçon N, Van Mechelen M, Morel S. 2014. Enhancement of adaptive immunity by the human vaccine adjuvant AS01 depends on activated dendritic cells. J Immunol 193:1920–1930. doi:10.4049/jimmunol.1400948 25024381

[77] Detienne S, Welsby I, Collignon C, Wouters S, Coccia M, Delhaye S, Van Maele L, Thomas S, Swertvaegher M, Detavernier A, Elouahabi A, Goriely S, Didierlaurent AM. 2016. Central role of CD169+ lymph node resident macrophages in the adjuvanticity of the QS-21 component of AS01. Sci Rep 6:39475. doi:10.1038/srep39475 27996000 PMC5172233

[78] Ho NI, Huis in ’t Veld LGM, Raaijmakers TK, Adema GJ. 2018. Adjuvants enhancing cross-presentation by dendritic cells: the key to more effective vaccines? Front Immunol 9. doi:10.3389/fimmu.2018.02874 PMC6300500 30619259

[79] Nair-Gupta P, Baccarini A, Tung N, Seyffer F, Florey O, Huang Y, Banerjee M, Overholtzer M, Roche PA, Tampé R, Brown BD, Amsen D, Whiteheart SW, Blander JM. 2014. TLR signals induce phagosomal MHC-I delivery from the endosomal recycling compartment to allow cross-presentation. Cell 158:506–521. doi:10.1016/j.cell.2014.04.054 25083866 PMC4212008

[80] Alloatti A, Kotsias F, Pauwels AM, Carpier JM, Jouve M, Timmerman E, Pace L, Vargas P, Maurin M, Gehrmann U, Joannas L, Vivar OI, Lennon-Duménil AM, Savina A, Gevaert K, Beyaert R, Hoffmann E, Amigorena S. 2015. Toll-like receptor 4 engagement on dendritic cells restrains phago-lysosome fusion and promotes cross-presentation of antigens. Immunity 43:1087–1100. doi:10.1016/j.immuni.2015.11.006 26682983

[81] Coccia M, Collignon C, Hervé C, Chalon A, Welsby I, Detienne S, van Helden MJ, Dutta S, Genito CJ, Waters NC, Van Deun K, Smilde AK, van den Berg RA, Franco D, Bourguignon P, Morel S, Garçon N, Lambrecht BN, Goriely S, van der Most R, Didierlaurent AM. 2017. Cellular and molecular synergy in AS01-adjuvanted vaccines results in an early IFNγ response promoting vaccine immunogenicity. NPJ Vaccines 2:25. doi:10.1038/s41541-017-0027-3 29263880 PMC5627273

[82] Cohen KW, De Rosa SC, Fulp WJ, deCamp AC, Fiore-Gartland A, Mahoney CR, Furth S, Donahue J, Whaley RE, Ballweber-Fleming L, et al.. 2023. A first-in-human germline-targeting HIV nanoparticle vaccine induced broad and publicly targeted helper T cell responses. Sci Transl Med 15:eadf3309. doi:10.1126/scitranslmed.adf3309 37224227 PMC11036875

[83] Shrivastava S, Carmen JM, Kim J, Peachman KK, Basu S, Alving R, Nettere D, Kundu G, Yum L, Rahman MA, et al.. 2025. ALFQ adjuvanted HIV-1 envelope protein vaccination elicits durable functional antibody and cellular responses in nonhuman primates. NPJ Vaccines 11:1. doi:10.1038/s41541-025-01322-7 41419739 PMC12764542

[84] Kim CG, Kye YC, Yun CH. 2019. The role of nanovaccine in cross-presentation of antigen-presenting cells for the activation of CD8^+^ T cell responses. Pharmaceutics 11:612. doi:10.3390/pharmaceutics11110612 31731667 PMC6920862

[85] Rock KL, Shen L. 2005. Cross-presentation: underlying mechanisms and role in immune surveillance. Immunol Rev 207:166–183. doi:10.1111/j.0105-2896.2005.00301.x 16181335

[86] Migueles SA, Nettere DM, Gavil NV, Wang LT, Toulmin SA, Kelly EP, Ward AJ, Lin S, Thompson SA, Peterson BA, et al.. 2023. HIV vaccines induce CD8(+) T cells with low antigen receptor sensitivity. Science 382:1270–1276. doi:10.1126/science.adg0514 38096385

[87] Andersen-Nissen E, Fiore-Gartland A, Ballweber Fleming L, Carpp LN, Naidoo AF, Harper MS, Voillet V, Grunenberg N, Laher F, Innes C, Bekker L-G, Kublin JG, Huang Y, Ferrari G, Tomaras GD, Gray G, Gilbert PB, McElrath MJ. 2021. Innate immune signatures to a partially-efficacious HIV vaccine predict correlates of HIV-1 infection risk. PLoS Pathog 17:e1009363. doi:10.1371/journal.ppat.1009363 33720973 PMC7959397

[88] Ko S-Y, Pegu A, Rudicell RS, Yang Z, Joyce MG, Chen X, Wang K, Bao S, Kraemer TD, Rath T, Zeng M, Schmidt SD, Todd J-P, Penzak SR, Saunders KO, Nason MC, Haase AT, Rao SS, Blumberg RS, Mascola JR, Nabel GJ. 2014. Enhanced neonatal Fc receptor function improves protection against primate SHIV infection. Nature 514:642–645. doi:10.1038/nature13612 25119033 PMC4433741

[89] Snow BJ, Keles NK, Grunst MW, Janaka SK, Behrens RT, Evans DT. 2024. Potent broadly neutralizing antibodies mediate efficient antibody-dependent phagocytosis of HIV-infected cells. PLoS Pathog 20:e1012665. doi:10.1371/journal.ppat.1012665 39466835 PMC11542898

[90] Chamcha V, Reddy PBJ, Kannanganat S, Wilkins C, Gangadhara S, Velu V, Green R, Law GL, Chang J, Bowen JR, Kozlowski PA, Lifton M, Santra S, Legere T, Chea LS, Chennareddi L, Yu T, Suthar MS, Silvestri G, Derdeyn CA, Gale M Jr, Villinger F, Hunter E, Amara RR. 2019. Strong T(H)1-biased CD4 T cell responses are associated with diminished SIV vaccine efficacy. Sci Transl Med 11. doi:10.1126/scitranslmed.aav1800 PMC7227795 31748228

[91] Kiepiela P, Ngumbela K, Thobakgale C, Ramduth D, Honeyborne I, Moodley E, Reddy S, de Pierres C, Mncube Z, Mkhwanazi N, et al.. 2007. CD8+ T-cell responses to different HIV proteins have discordant associations with viral load. Nat Med 13:46–53. doi:10.1038/nm1520 17173051

[92] Carnathan DG, Wetzel KS, Yu J, Lee ST, Johnson BA, Paiardini M, Yan J, Morrow MP, Sardesai NY, Weiner DB, Ertl HCJ, Silvestri G. 2015. Activated CD4+CCR5+ T cells in the rectum predict increased SIV acquisition in SIVGag/Tat-vaccinated rhesus macaques. Proc Natl Acad Sci USA 112:518–523. doi:10.1073/pnas.1407466112 25550504 PMC4299179

[93] Douek DC, Brenchley JM, Betts MR, Ambrozak DR, Hill BJ, Okamoto Y, Casazza JP, Kuruppu J, Kunstman K, Wolinsky S, Grossman Z, Dybul M, Oxenius A, Price DA, Connors M, Koup RA. 2002. HIV preferentially infects HIV-specific CD4+ T cells. Nature 417:95–98. doi:10.1038/417095a 11986671

[94] Parrish NF, Gao F, Li H, Giorgi EE, Barbian HJ, Parrish EH, Zajic L, Iyer SS, Decker JM, Kumar A, et al.. 2013. Phenotypic properties of transmitted founder HIV-1. Proc Natl Acad Sci USA 110:6626–6633. doi:10.1073/pnas.1304288110 23542380 PMC3637789

[95] Saunders KO, Edwards RJ, Tilahun K, Manne K, Lu X, Cain DW, Wiehe K, Williams WB, Mansouri K, Hernandez GE, et al.. 2022. Stabilized HIV-1 envelope immunization induces neutralizing antibodies to the CD4bs and protects macaques against mucosal infection. Sci Transl Med 14:eabo5598. doi:10.1126/scitranslmed.abo5598 36070369 PMC10034035

[96] Mielke D, Tuyishime M, Kelkar NS, Wang Y, Parks R, Santra S, Rountree W, Williams LD, Peters T, Eisel N, et al.. 2025. Computationally selected multivalent HIV-1 subtype C vaccine protects against heterologous SHIV challenge. Vaccines (Basel) 13:231. doi:10.3390/vaccines13030231 40266065 PMC11945704

[97] Beck Z, Matyas GR, Jalah R, Rao M, Polonis VR, Alving CR. 2015. Differential immune responses to HIV-1 envelope protein induced by liposomal adjuvant formulations containing monophosphoryl lipid A with or without QS21. Vaccine 33:5578–5587. doi:10.1016/j.vaccine.2015.09.001 26372857

[98] Beck Z, Torres OB, Matyas GR, Lanar DE, Alving CR. 2018. Immune response to antigen adsorbed to aluminum hydroxide particles: effects of co-adsorption of ALF or ALFQ adjuvant to the aluminum-antigen complex. J Control Release 275:12–19. doi:10.1016/j.jconrel.2018.02.006 29432824 PMC5878139

[99] Rao M, Onkar S, Peachman KK, White Y, Trinh HV, Jobe O, Zhou Y, Dawson P, Eller MA, Matyas GR, Alving CR. 2018. Liposome-encapsulated human immunodeficiency virus-1 gp120 induces potent V1V2-specific antibodies in humans. J Infect Dis 218:1541–1550. doi:10.1093/infdis/jiy348 29893872

[100] Seaton KE, Ballweber L, Lan A, Donathan M, Hughes S, Vojtech L, Moody MA, Liao H-X, Haynes BF, Galloway CG, Richardson BA, Karim SA, Dezzutti CS, McElrath MJ, Tomaras GD, Hladik F. 2014. HIV-1 specific IgA detected in vaginal secretions of HIV uninfected women participating in a microbicide trial in Southern Africa are primarily directed toward gp120 and gp140 specificities. PLoS One 9:e101863. doi:10.1371/journal.pone.0101863 25054205 PMC4108330

[101] Tomaras GD, Yates NL, Liu P, Qin L, Fouda GG, Chavez LL, Decamp AC, Parks RJ, Ashley VC, Lucas JT, et al.. 2008. Initial B-cell responses to transmitted human immunodeficiency virus type 1: virion-binding immunoglobulin M (IgM) and IgG antibodies followed by plasma anti-gp41 antibodies with ineffective control of initial viremia. J Virol 82:12449–12463. doi:10.1128/JVI.01708-08 18842730 PMC2593361

[102] Yates NL, deCamp AC, Korber BT, Liao H-X, Irene C, Pinter A, Peacock J, Harris LJ, Sawant S, Hraber P, Shen X, Rerks-Ngarm S, Pitisuttithum P, Nitayapan S, Berman PW, Robb ML, Pantaleo G, Zolla-Pazner S, Haynes BF, Alam SM, Montefiori DC, Tomaras GD. 2018. HIV-1 envelope glycoproteins from diverse clades differentiate antibody responses and durability among vaccinees. J Virol 92. doi:10.1128/JVI.01843-17 PMC5874409 29386288

[103] Pinter A, Honnen WJ, Kayman SC, Trochev O, Wu Z. 1998. Potent neutralization of primary HIV-1 isolates by antibodies directed against epitopes present in the V1/V2 domain of HIV-1 gp120. Vaccine 16:1803–1811. doi:10.1016/s0264-410x(98)00182-0 9795384

[104] Brown EP, Licht AF, Dugast AS, Choi I, Bailey-Kellogg C, Alter G, Ackerman ME. 2012. High-throughput, multiplexed IgG subclassing of antigen-specific antibodies from clinical samples. J Immunol Methods 386:117–123. doi:10.1016/j.jim.2012.09.007 23023091 PMC3475184

[105] Mdluli T, Jian N, Slike B, Paquin-Proulx D, Donofrio G, Alrubayyi A, Gift S, Grande R, Bryson M, Lee A, et al.. 2020. RV144 HIV-1 vaccination impacts post-infection antibody responses. PLoS Pathog 16:e1009101. doi:10.1371/journal.ppat.1009101 33290394 PMC7748270

[106] Montefiori DC. 2009. Measuring HIV neutralization in a luciferase reporter gene assay. Methods Mol Biol 485:395–405. doi:10.1007/978-1-59745-170-3_26 19020839

[107] Ackerman ME, Moldt B, Wyatt RT, Dugast AS, McAndrew E, Tsoukas S, Jost S, Berger CT, Sciaranghella G, Liu Q, Irvine DJ, Burton DR, Alter G. 2011. A robust, high-throughput assay to determine the phagocytic activity of clinical antibody samples. J Immunol Methods 366:8–19. doi:10.1016/j.jim.2010.12.016 21192942 PMC3050993

[108] Minh BQ, Hahn MW, Lanfear R. 2020. New methods to calculate concordance factors for phylogenomic datasets. Mol Biol Evol 37:2727–2733. doi:10.1093/molbev/msaa106 32365179 PMC7475031

[109] Schliep KP. 2011. Phangorn: phylogenetic analysis in R. Bioinformatics 27:592–593. doi:10.1093/bioinformatics/btq706 21169378 PMC3035803

[110] Roederer M, Nozzi JL, Nason MC. 2011. SPICE: exploration and analysis of post-cytometric complex multivariate datasets. Cytometry A 79:167–174. doi:10.1002/cyto.a.21015 21265010 PMC3072288

[111] Richard J, Veillette M, Brassard N, Iyer SS, Roger M, Martin L, Pazgier M, Schön A, Freire E, Routy J-P, Smith AB III, Park J, Jones DM, Courter JR, Melillo BN, Kaufmann DE, Hahn BH, Permar SR, Haynes BF, Madani N, Sodroski JG, Finzi A. 2015. CD4 mimetics sensitize HIV-1-infected cells to ADCC. Proc Natl Acad Sci USA 112:E2687–94. doi:10.1073/pnas.1506755112 25941367 PMC4443331

[112] Fontaine J, Chagnon-Choquet J, Valcke HS, Poudrier J, Roger M. 2011. High expression levels of B lymphocyte stimulator (BLyS) by dendritic cells correlate with HIV-related B-cell disease progression in humans. Blood 117:145–155. doi:10.1182/blood-2010-08-301887 20870901

[113] Fontaine J, Coutlée F, Tremblay C, Routy J-P, Poudrier J, Roger M. 2009. HIV infection affects blood myeloid dendritic cells after successful therapy and despite nonprogressing clinical disease. J Infect Dis 199:1007–1018. doi:10.1086/597278 19231994

