# Supplementary material for: Rapid and robust immune response boosting with potent, next-generation adjuvant in viral vector-primed primates
Source: J Virol. 2026 Jul 27;100(8):e00389-26. doi: 10.1128/jvi.00389-26 (PMC13483363; doi:10.1128/jvi.00389-26)
Supplement: Supplementary file 1 [file jvi.00389-26-s0001.pdf]

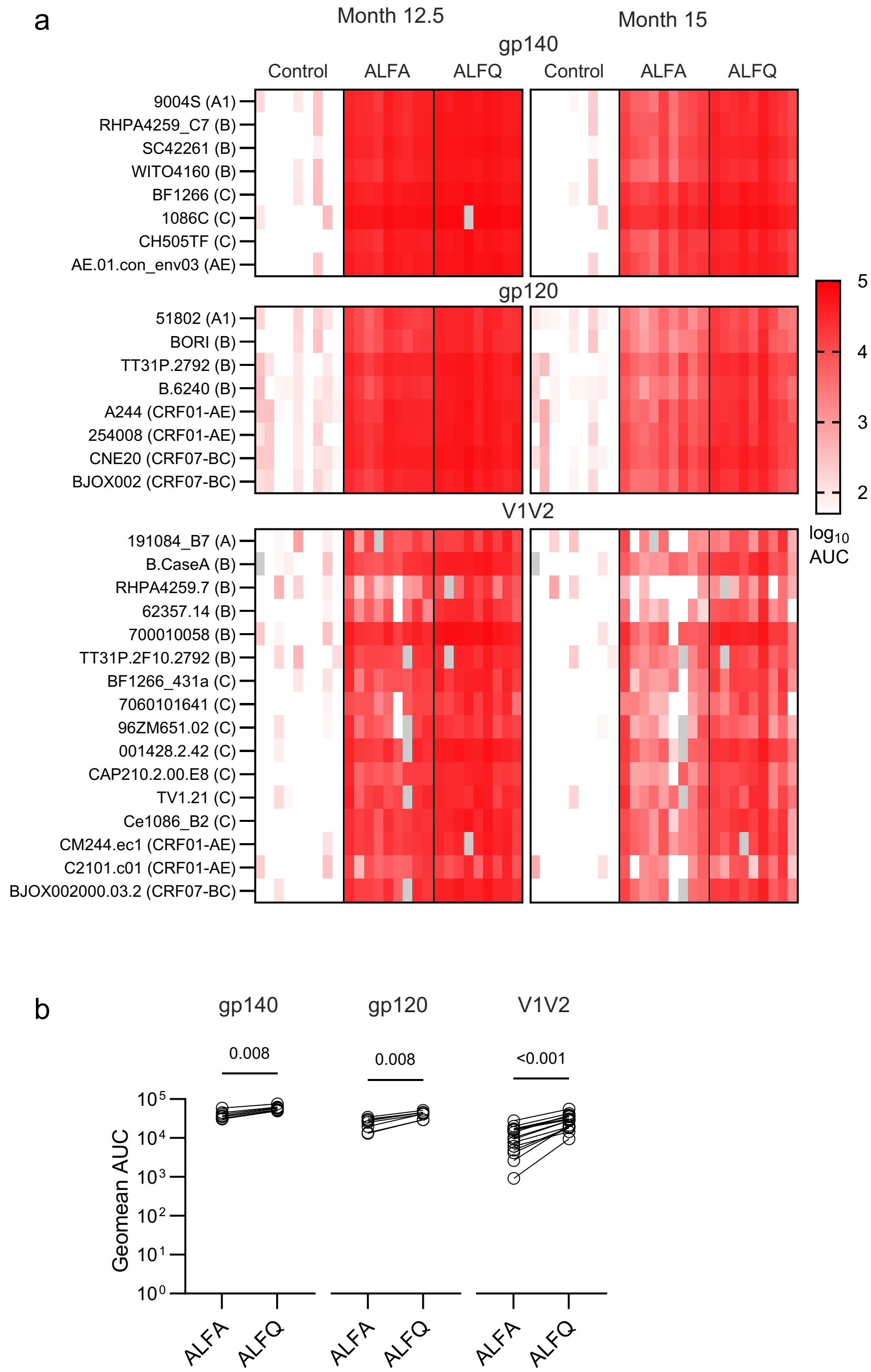

Fig S1. Env-specific serum binding antibody responses to additional Env antigens. Env-specific IgG responses to HIV-1 antigens in serum two weeks following the fourth immunization (month 12.5) and at the time of challenge (month 15) were measured by BAMA. **(a)** Responses are shown as area under the curve (AUC) for the indicated strains for gp140, gp120, gp41 and gp70-scaffolded V1V2 antigens. Each column represents an individual animal. Grey boxes indicate values excluded due to high background at baseline. **(b)** Paired comparison of group geometric mean AUC for each of the gp140, gp120 and V1V2 antigens at the month 12.5 timepoint. P values reflect paired Wilcoxon test.

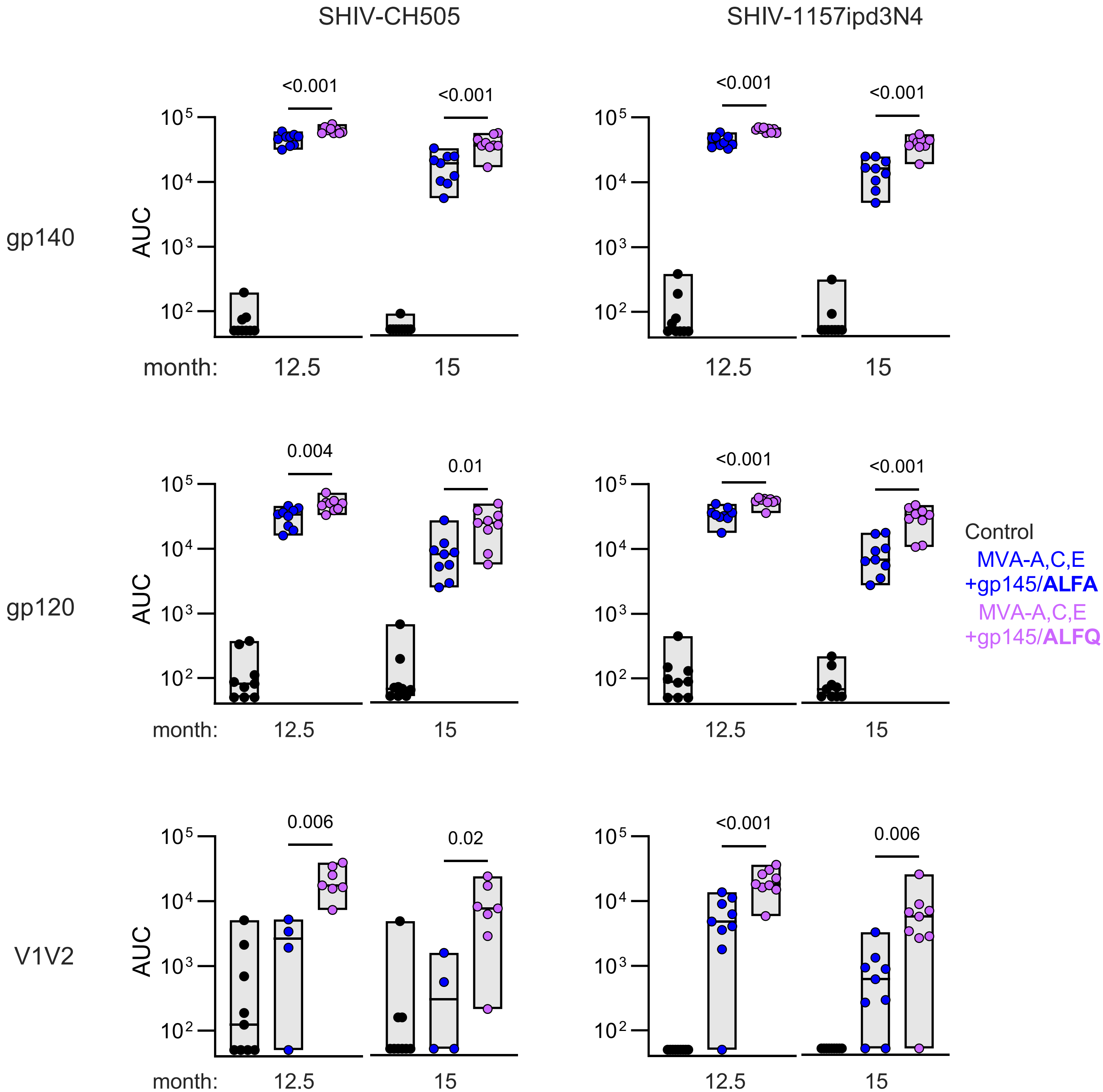

Fig S2. Env-specific serum binding antibody responses to selected clade C Env antigens. Env-specific IgG responses to selected clade C Env antigens (SHIV-CH505 and SHIV-1157ipd3N4) in serum two weeks following the fourth immunization (month 12.5) and at the time of challenge (month 15) were measured by BAMA. Responses are shown as area under the curve (AUC). Gray bars reflect range; black lines depict medians; *P* values in reflect Mann-Whitney test comparing ALFA and ALFQ-vaccinated groups. Symbol colors reflect control (black), ALFA (blue), and ALFQ (purple) groups.

		Month 12.5				Month 15			
**Group**	**Animal ID**	**MLV**	**6980.v0.c22**	**SHIV-CH505.375H**	**SHIV-1157ipd3N4**	**MLV**	**6980.v0.c22**	**SHIV-CH505.375H**	**SHIV-1157ipd3N4**
Control	CO77	<20	<20	<20	<20	<20	<20	<20	<20
	CO78	<20	<20	<20	<20	<20	<20	<20	**21**
	CO98	<20	<20	<20	<20	<20	<20	25	<20
	CP09	<20	<20	<20	<20	<20	23	<20	<20
	CK25	<20	<20	<20	<20	<20	<20	<20	<20
	CK43	<20	<20	<20	<20	<20	<20	<20	<20
	CL46	<20	<20	<20	<20	<20	<20	<20	<20
	CL59	<20	<20	<20	<20	<20	<20	<20	<20
	CO68	44	64	41	45	52	74	49	47
MVA-A,C,E +gp145/ALFA	CI41	<20	<20	<20	<20	<20	<20	<20	<20
	CK65	<20	<20	<20	<20	<20	<20	<20	<20
	CL35	<20	<20	<20	<20	<20	<20	<20	<20
	CM95	<20	<20	<20	<20	<20	<20	<20	<20
	CO10	<20	<20	<20	<20	<20	<20	<20	<20
	CO56	<20	<20	<20	<20	<20	<20	<20	<20
	CO88	<20	<20	<20	<20	<20	<20	<20	<20
	CO97	<20	<20	<20	<20	<20	<20	<20	<20
	CO99	<20	<20	<20	<20	<20	<20	<20	<20
MVA-A,C,E +gp145/ALFQ	CI67	<20	<20	<20	<20	<20	<20	<20	<20
	CK24	<20	<20	<20	<20	<20	20	<20	<20
	CL31	<20	<20	<20	<20	<20	<20	<20	<20
	CO05	<20	<20	<20	<20	<20	<20	<20	24
	CO36	<20	<20	<20	<20	<20	<20	<20	<20
	CK60	<20	<20	<20	<20	<20	<20	<20	<20
	CL19	<20	<20	<20	<20	<20	<20	<20	<20
	CO72	<20	<20	<20	<20	<20	<20	<20	<20
	CO74	<20	<20	<20	<20	<20	<20	<20	<20

Fig S3. Vaccine-elicited neutralizing antibody responses. Pseudovirus neutralization by vaccinated animal sera was measured in TZM-bl target cells at two weeks following the fourth immunization (month 12.5) and at the time of challenge (month 15). Pseudoviruses expressing the vaccine Env 6980.v0.c22, and two selected clade C Env SHIV-CH505.375H and SHIC-1157ipd3N4 were measured, in addition to an MLV negative control. The serum dilution that resulted in 50% inhibition (ID50) is reported for each animal by vaccine group.

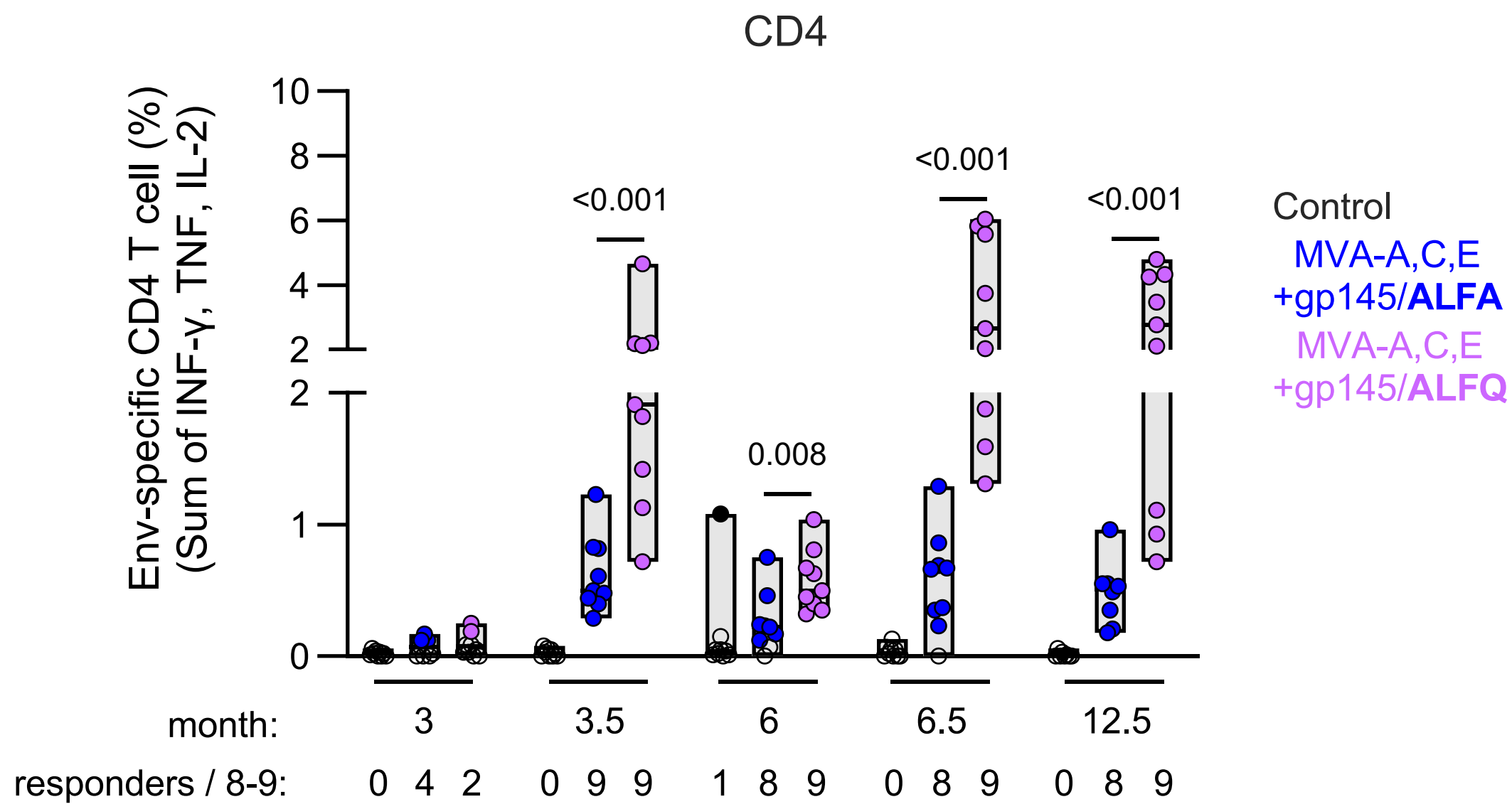

Fig S4. Longitudinal Env-specific CD4 T cell responses spanning month 3 to month 12.5. PBMC HIV-1 Env-specific CD4 T cells were measured longitudinally by ICS for IFNγ, TNFα and IL-2. The frequency of memory cells that expressed one or more cytokines following *ex vivo* Env PTE peptide pool stimulation is shown for each animal by arm (one pool of 467 peptides, lot 180433). Boolean combinations of cytokine-positive memory T cell responses were summed. Positive responses, defined as greater than three times the group background at baseline are depicted as closed symbols and the positive rate within each group is indicated below each graph. Gray bars reflect range; black lines depict means; *P* values reflect Mann-Whitney test comparing the two vaccine arms. Symbol colors reflect control (black), ALFA (blue), and ALFQ (purple) groups.

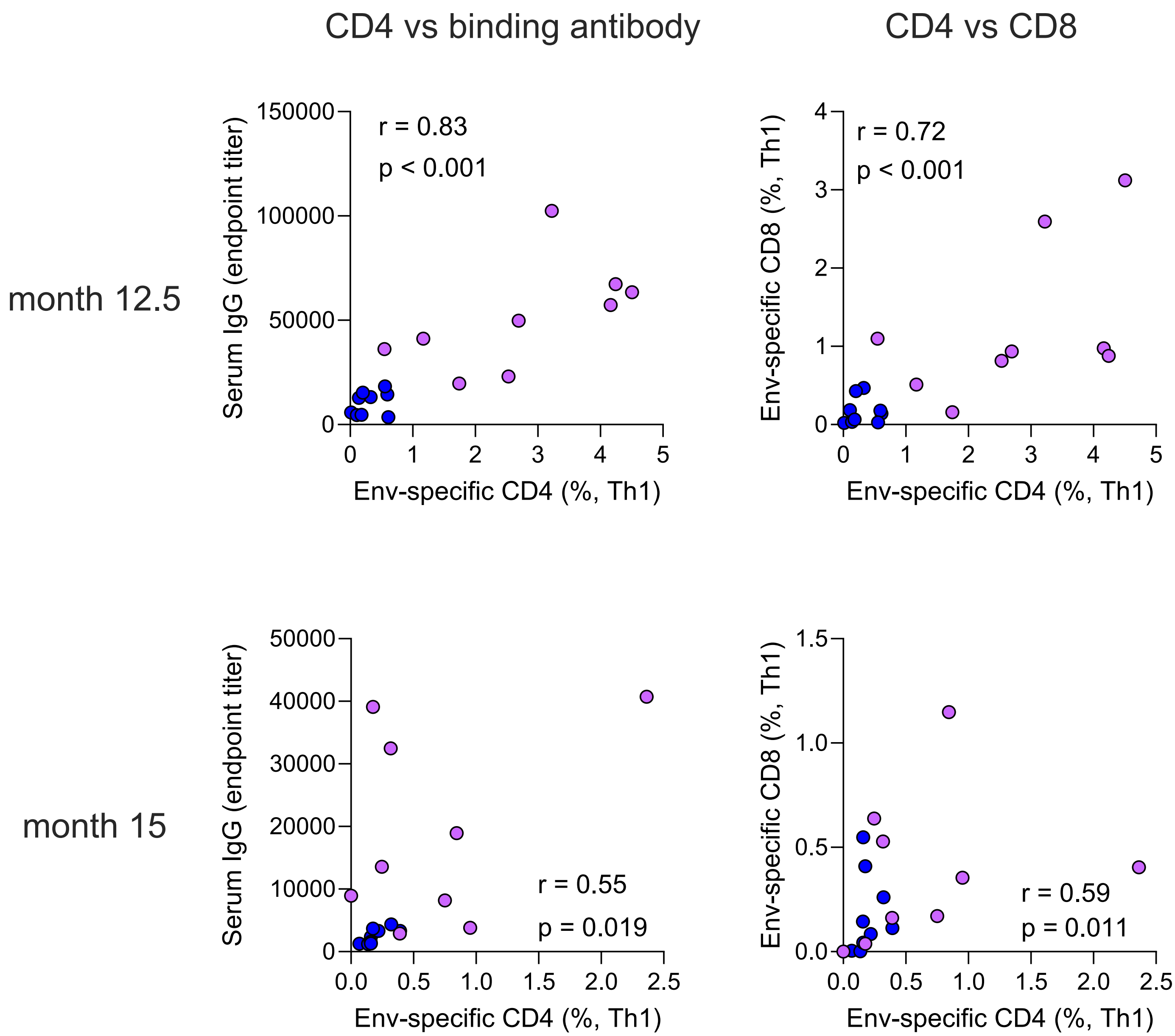

Fig S5. Immune response correlates. Bivariate plots depicting association between vaccine-matched adaptive immune responses – CD4 and CD8 T cell responses measured by ICS (Th1 cytokines – IFNγ, TNFα, IL-2) and gp145 binding antibody endpoint titers. Spearman correlation rho values and unadjusted p values are overlaid on the graphs. Purple symbols represent ALFQ-vaccinated animals, blue symbols represent ALFA-vaccinated animals.

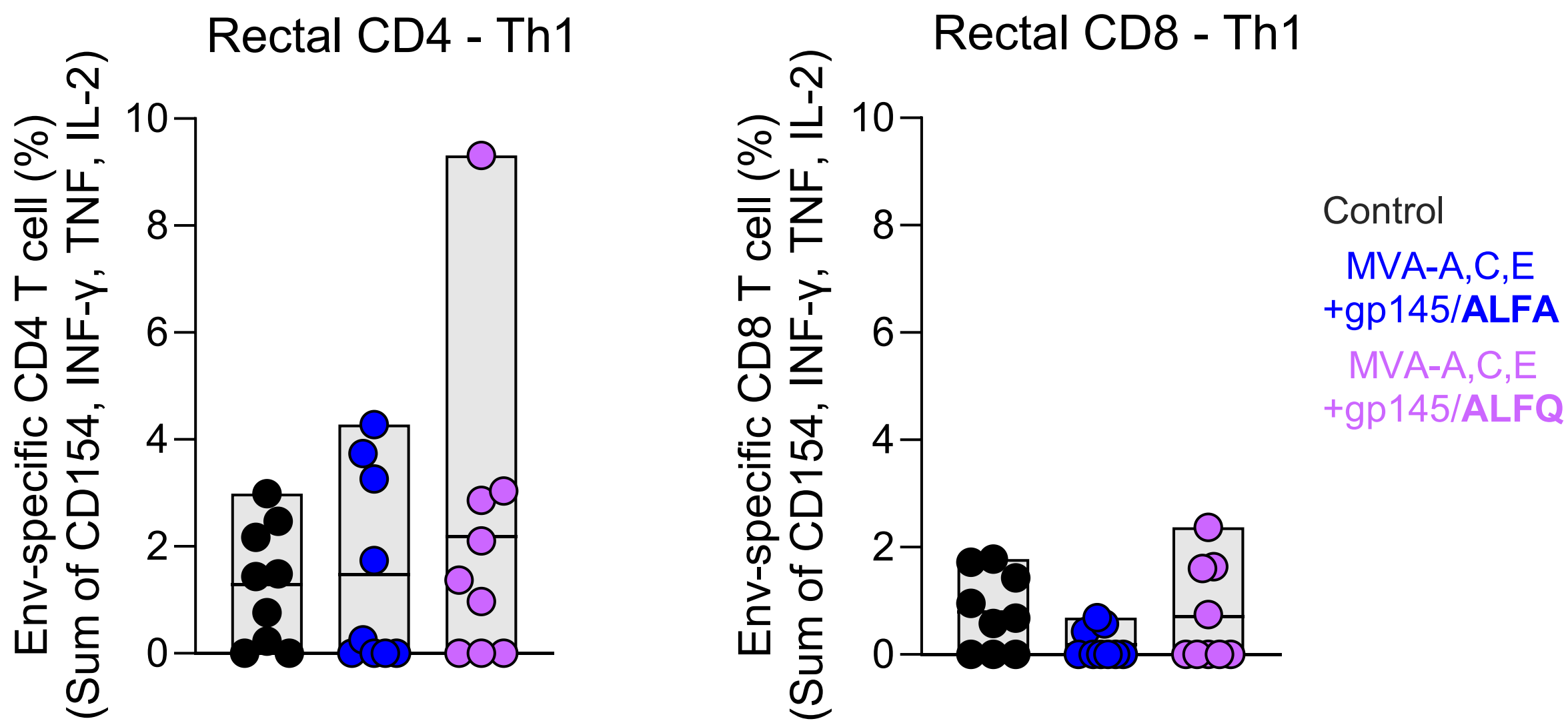

Fig S6. Rectal mucosa HIV-specific CD4 and CD8 T cell responses. HIV-1 Env-specific CD4 and CD8 T cells isolated from rectal pinch biopsies were measured by ICS for CD154, IFNγ, TNFα and IL-2 two weeks following the third immunization (month 6.5) The frequency of memory cells that expressed one or more cytokines following *ex vivo* Env PTE peptide pool stimulation is shown for each animal by arm. Boolean combinations of cytokine-positive memory T cell responses were summed. Gray bars depict range; black lines depict means. Mann-Whitney test was performed comparing the two active arms, no significant differences were found. Symbol colors reflect control (black), ALFA (blue), and ALFQ (purple) groups.

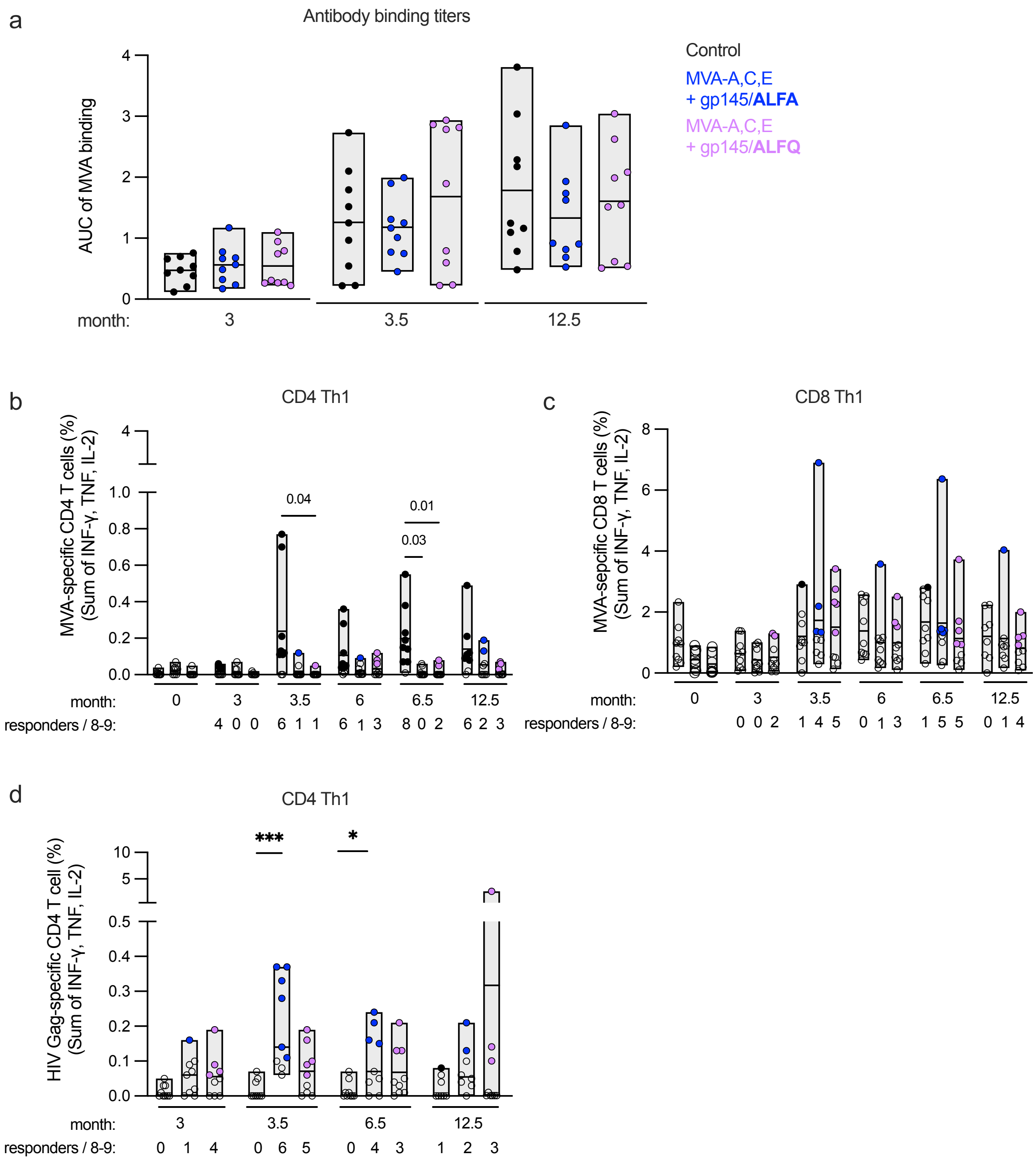

Fig S7. Humoral and cellular responses specific to the MVA vector. (A) Serum IgG binding antibody responses to vaccinia virus D8L protein are shown following the first (month 3), second (month 3.5) and fourth (month 12.5) vaccinations. Data is displayed as the AUC of ELISA binding to the vaccinia antigen. PBMC MVA-specific CD4 (B), CD8 (C) T cells and HIV Gag-specific CD4 (D) T cells were measured by ICS for IFNγ, TNFα and IL-2 longitudinally. The frequency of memory cells that expressed one or more cytokines following MVA co-culture or HIV Gag peptide stimulation is shown for each animal by arm. Boolean combinations of cytokine-positive memory T cell responses were summed. Positive responses, defined as greater than three times the group background at baseline are depicted as closed symbols and the positive rate within each group is indicated below each graph. Gray bars depict range; black lines depict means. *P* values reflect Kruskal-Wallis test followed by a Dunn's posttest. Symbol colors reflect control (black), ALFA (blue), and ALFQ (purple) groups.

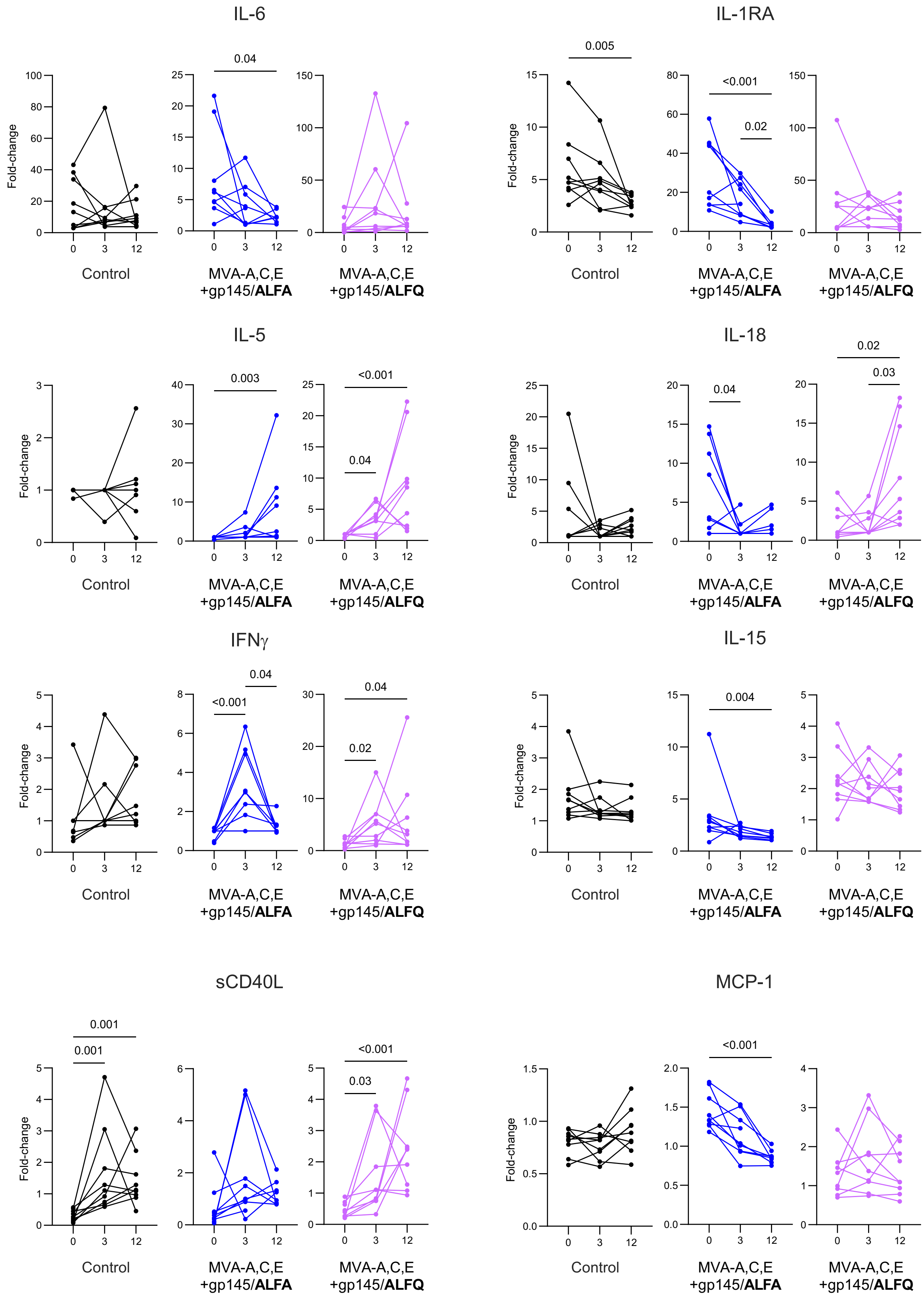

Fig S8. Longitudinal vaccine-elicited plasma cytokines. Elicitation of individual plasma cytokines compared within each group following vaccinations 1, 2 and 4 (months 0, 3 and 12). Cytokine elicitation is expressed as fold-change between the time of vaccination and 24 hours. *P* values reflect Kruskal-Wallis test followed by Dunn's posttest. Cytokines in which significant differences (p < 0.05) were found in one or more groups are shown.

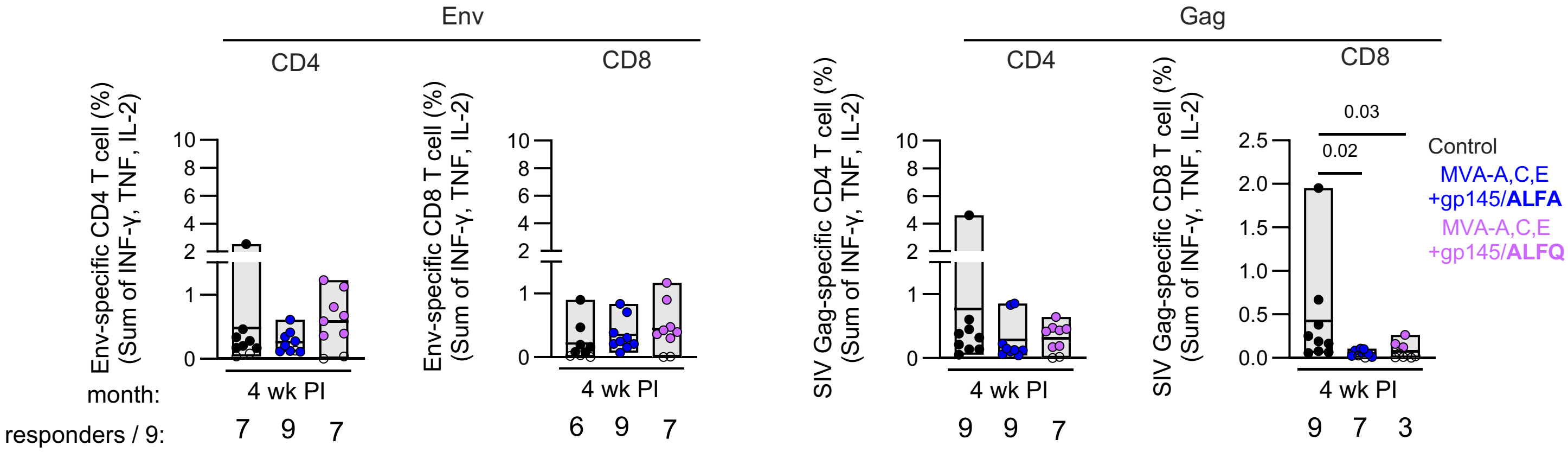

Fig S9. Post-infection Env- and SIV Gag-specific T cell responses. PBMC HIV-1 Env-specific (left) and SIV-1 Gag-specific (right) CD4 and CD8 T cells were measured by ICS for IFNγ, TNFα and IL-2 four weeks following SHIV acquisition. The frequency of memory cells expressing one or more cytokines following *ex vivo* Env PTE or SIV Gag peptide pool stimulation is shown for each animal by arm. Boolean combinations of cytokine-positive memory T cell responses were summed. Positive responses, defined as greater than three times the group background at baseline are depicted as closed symbols and the positive rate within each group is indicated below each graph. Gray bars reflect range; black lines depict means; *P* values reflect Kruskal-Wallis test followed by a Dunn's posttest test. Symbol colors reflect control (black), ALFA (blue), and ALFQ (purple) groups.

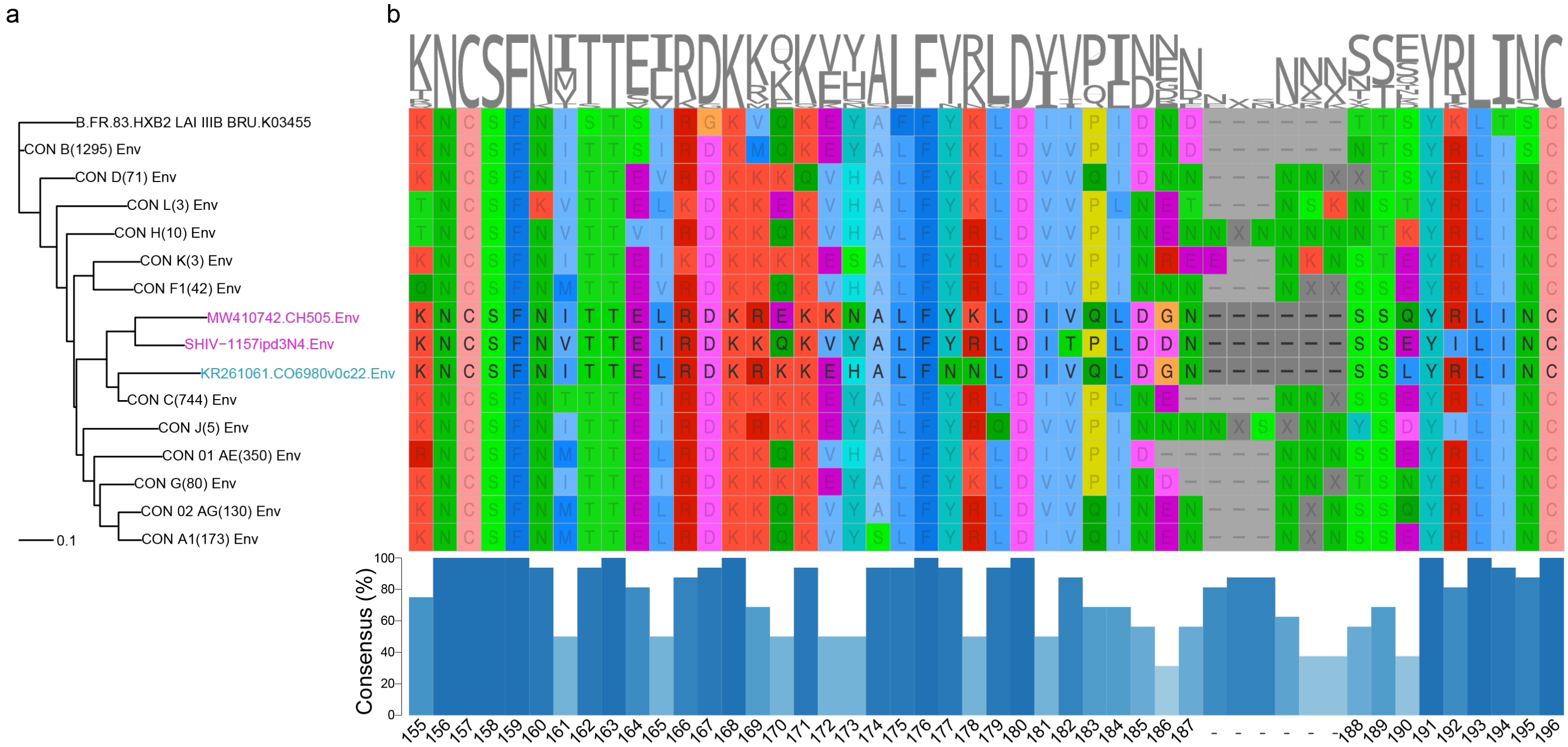

Fig S10. Comparison of Env sequences between vaccine and challenge strains. (**A**) Tree showing genetic relatedness of Env sequences from the vaccine strain (C06980v0c22) with SHIV-CH505 and SHIV-1157ipd3N4 along with a panel of representative clade C Envs. (**B**) Logo plot and sequence alignment of the V2 region for each of the HIV-1 strains depicted in the tree in (A). Bar graph (bottom) shows the percentage of sequences at each site with the consensus residue. Sites refer to HXB2 numbering.

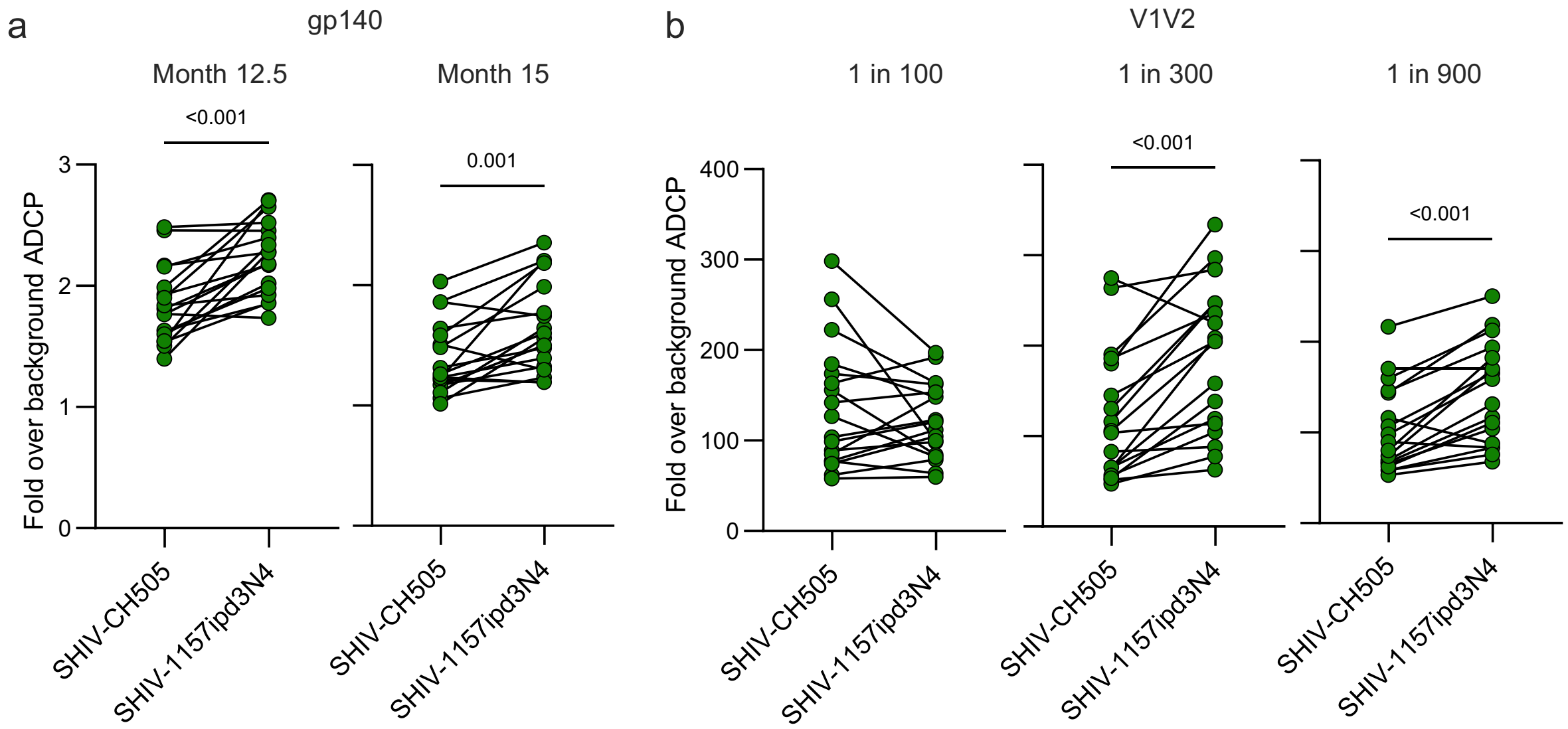

Fig S11. Comparison of Env-specific ADCP responses to CH505 and 1157ipd3N4 Env antigens. ADCP measured against gp140 (**A**) and V1V2 (**B**) SHIV-CH505 and 1157ipd3N4. ADCP to gp140 antigens was performed at a 1:1,000 plasma dilution; month 12.5 V1V2 responses were assessed at the three indicated dilutions. Data were normalized as fold-change over negative control wells (no sera). Significance was assessed using a paired Wilcoxon test.

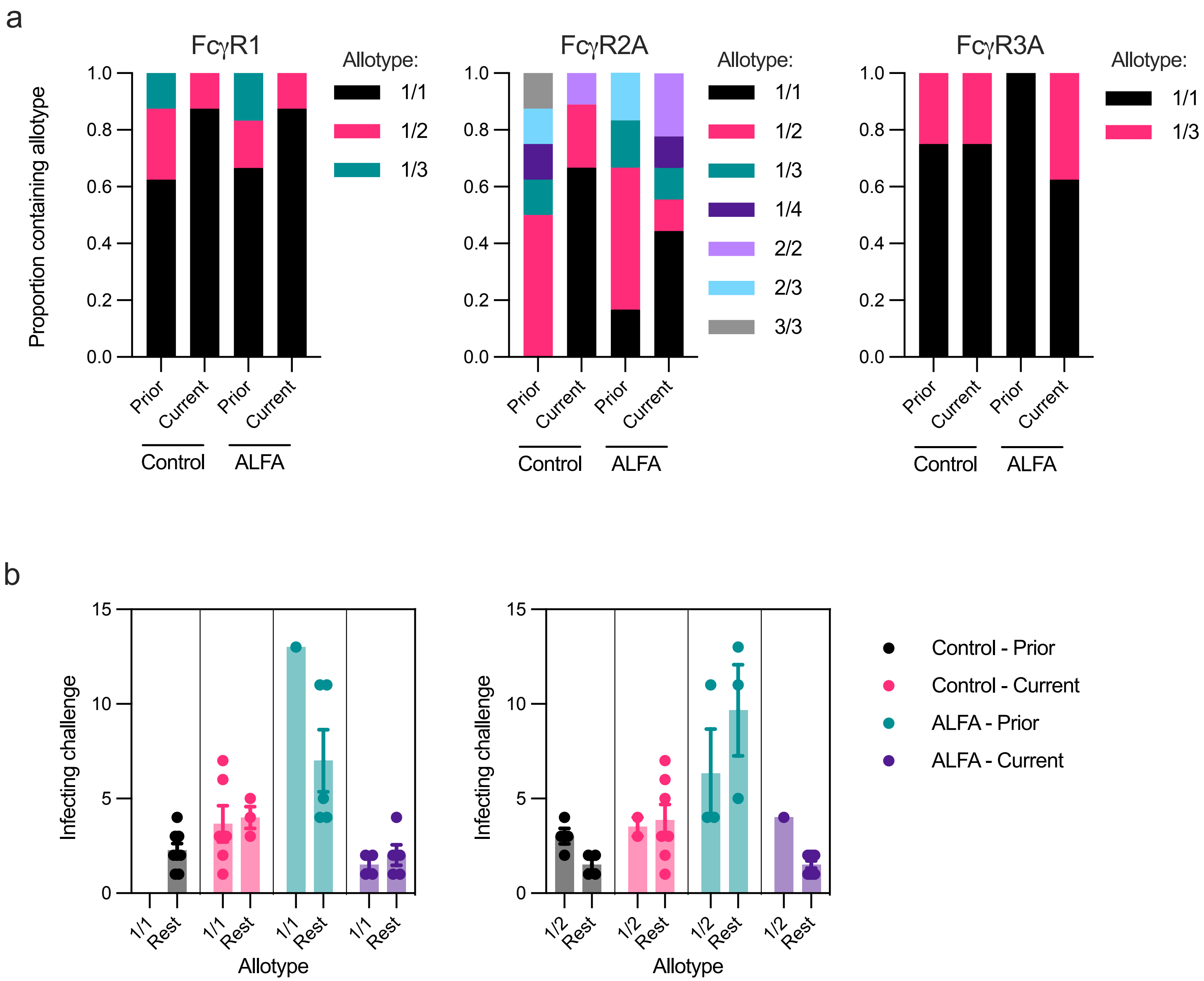

Fig S12. FcγR allotype comparison between groups. (**A**) Allotypes of FcγR1, FcγR2A and FcγR3A in control and ALFA-vaccinated groups from the present study and that reported in Om et al. Animals may be homozygous or heterozygous for each allotype. (**B**) Time to infection by FcγR2A allotype. Animals within each group expressing either the 1/1 (left) or 1/2 (right) FcγR2A allotypes are compared to animals expressing any other FcγR2A allotype with respect to the number of challenges to SHIV acquisition. Significance was assessed for each group using a Mann-Whitney test, no significant differences were seen.

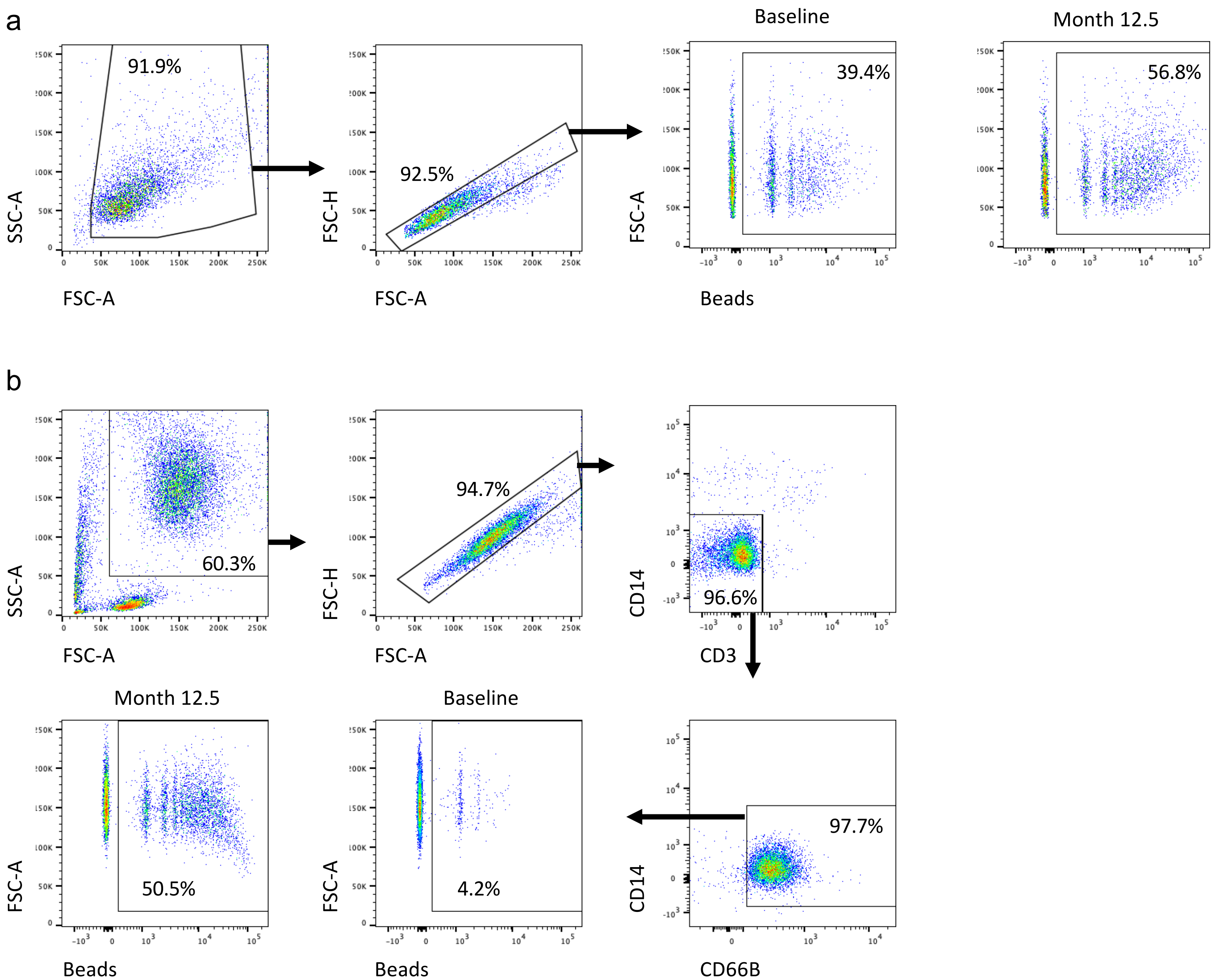

Fig S13. Representative gating strategy for ADCP and ADNP responses. Flow cytometry gating applied to representative samples for ADCP (A) or ADNP (B). ADCP sequential gates were applied from left to right to identify THP-1 cells positive for antigen-coated beads. ADNP sequential gating identifies bead-positive neutrophils (SSC high, CD3-, CD14-, CD66B+).

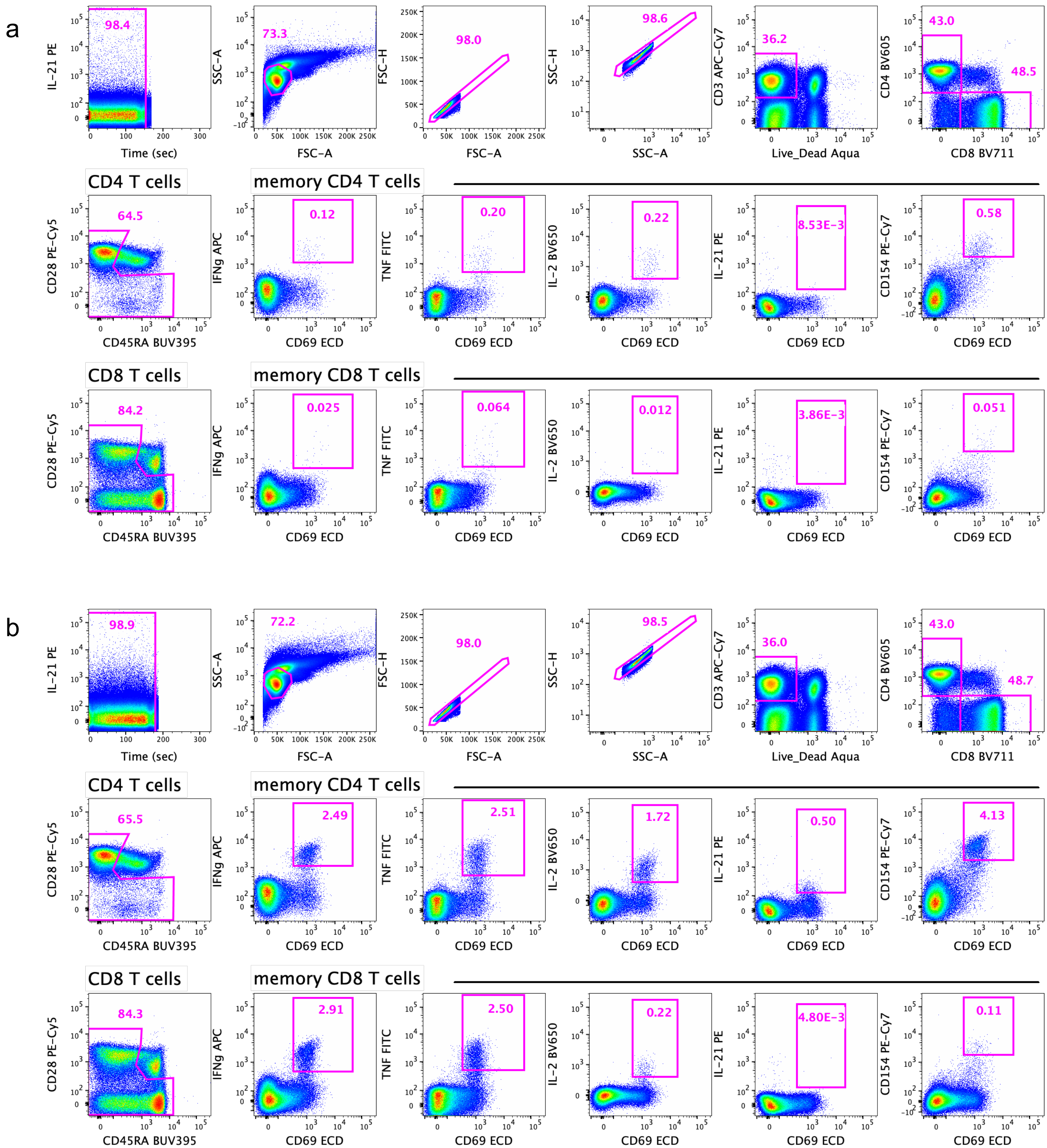

Fig S14. Representative flow cytometry staining and gating strategy for PBMC T cell responses. Flow cytometry gating applied to macaque PBMC T cell ICS data. FACS plots depict staining following stimulation with DMSO (A) or PTE Env peptide pool (B) for a representative ALFQ-adjuvanted animal at study month 6.5. Sequential gates were applied from left to right (top row) to identify CD4+ and CD8+ T cells. Cytokine production was measured in total memory CD4+ (middle) and CD8+ (bottom) T cells by excluding the naïve population (CD28+ CD45RA+). Cytokine-positive cells were identified by co-expression of CD69.

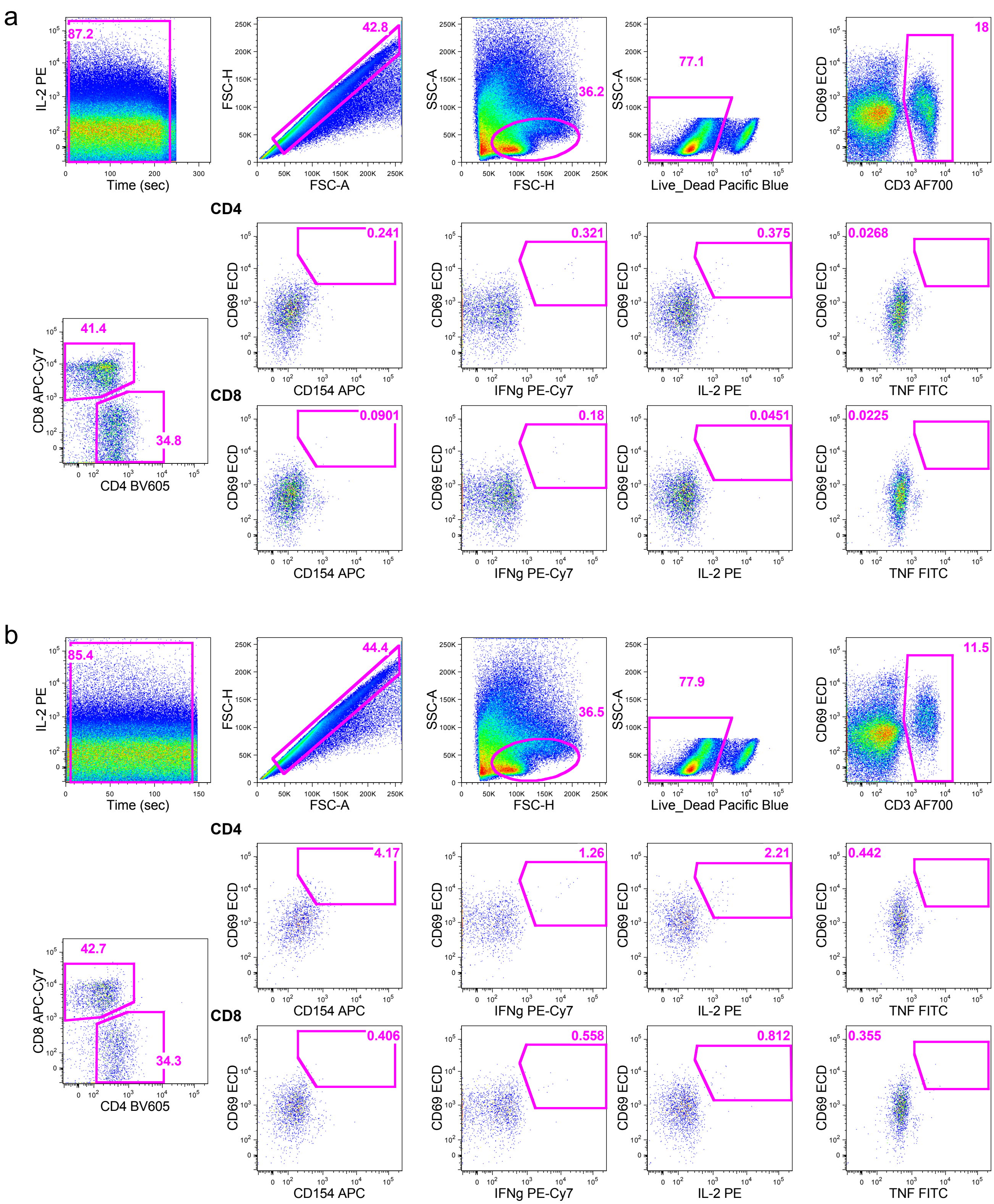

Fig S15. Representative flow cytometry staining and gating strategy for rectal T cell responses. Flow cytometry gating applied to macaque rectal T cell ICS data. FACS plots depict staining following stimulation with DMSO (A) or PTE Env peptide pool (B) for a representative ALFQ-adjuvanted animal at study month 6.5. Sequential gates were applied from left to right (top row) to identify CD4+ and CD8+ T cells. Cytokine production was measured in total CD4+ (middle) and CD8+ (bottom) T cells. Cytokine-positive cells were identified by co-expression of CD69.
